# The GAGA factor regulatory network: Identification of GAGA factor associated proteins

**DOI:** 10.1371/journal.pone.0173602

**Published:** 2017-03-15

**Authors:** Dmitry Lomaev, Anna Mikhailova, Maksim Erokhin, Alexander V. Shaposhnikov, James J. Moresco, Tatiana Blokhina, Daniel Wolle, Tsutomu Aoki, Vladimir Ryabykh, John R. Yates, Yulii V. Shidlovskii, Pavel Georgiev, Paul Schedl, Darya Chetverina

**Affiliations:** 1 Institute of Gene Biology, Russian Academy of Sciences, Moscow, Russia; 2 Department of Chemical Physiology, SR302B, The Scripps Research Institute, La Jolla, California, United States of America; 3 Department of Molecular Biology Princeton University, Princeton, NJ, United States of America; 4 Institute of Animal Physiology, Biochemistry and Nutrition, Borovsk, Russia; Centre National de la Recherche Scientifique, FRANCE

## Abstract

The Drosophila GAGA factor (GAF) has an extraordinarily diverse set of functions that include the activation and silencing of gene expression, nucleosome organization and remodeling, higher order chromosome architecture and mitosis. One hypothesis that could account for these diverse activities is that GAF is able to interact with partners that have specific and dedicated functions. To test this possibility we used affinity purification coupled with high throughput mass spectrometry to identify GAF associated partners. Consistent with this hypothesis the GAF interacting network includes a large collection of factors and complexes that have been implicated in many different aspects of gene activity, chromosome structure and function. Moreover, we show that GAF interactions with a small subset of partners is direct; however for many others the interactions could be indirect, and depend upon intermediates that serve to diversify the functional capabilities of the GAF protein.

## Introduction

The *Drosophila* GAGA factor (GAF) is an unusually versatile DNA binding protein that functions in remarkably diverse range of regulatory contexts. GAF was first identified as a transcriptional activator in *in vitro* transcription experiments with the *Ultrabithorax (Ubx)* and *engrailed (en)* genes. It bound to GAGAG motifs in the promoter region and stimulated transcription [1–3]. Consistent with a function in transcriptional activation, mutations in the gene encoding GAF, *Trithorax-like* (*Trl*), were shown to dominantly enhance the haploinsufficiency of the *Ubx* gene [4]. Moreover, the *Trl* mutations also dominantly enhanced position effect variegation (PEV) [4]. While these findings suggested that GAF functions as a conventional transcriptional activator, *in vitro* chromatin assembly experiments pointed to a rather different and unexpected role. When GAF was included in chromatin assembly assays using a plasmid containing the *hsp70* gene as the DNA template, it was found to mediate the formation of a nucleosome free region spanning the GAF binding motifs in the *hsp70* promoter [5]. The GAF factor helped recruit chromatin remodeling complexes to the template, and then functioned to exclude nucleosomes from the exposed promoter sequence [6]. Amongst the remodeling complexes that are thought to function together with GAF are PBAP, NURF and FACT [7–10]. A role in the formation/maintenance of nucleosome free regions of chromatin *in vitro* is recapitulated *in vivo* in transgene experiments with the *hsp26* and *hsp70* genes [11,12]. In addition to ensuring that promoter sequences are accessible, GAF is thought to play a more direct role in transcription by regulating promoter pausing [13–15]. These are not, however, the only known biological activities of the GAF protein. It has also been implicated in Polycomb group (PcG) dependent silencing [16–18], chromosome condensation and segregation during mitosis [19] and boundary activity [20]. Consistent with these multiple functions, GAF binding sequences are found in promoters, enhancers, Polycomb response elements (PREs) and boundary elements, while chromatin immunoprecipitation experiments localize GAF protein to these elements *in vivo* [21–26].

It is not yet understood how GAF carries out this diverse array of functions. The GAF protein itself has a relatively simple structure. It has an N-terminal BTB/POZ domain, a central C2H2-type zinc finger and several alternative glutamine rich (Q) C-terminal domains. The single zinc finger domain is responsible for DNA binding to the GAGAG pentanucleotide [27]. As there is little apparent flexibility in the DNA recognition properties of GAF, a plausible idea is that its different activities depend upon the ability of the GAF protein to interact either directly or indirectly with multiple partners. There is already evidence supporting this possibility. The GAF BTB/POZ domain has been shown to mediate protein-protein interactions and it participates in the formation of homo-oligomers and hetero-oligomers with other BTB/POZ proteins [28–30]. These BTB/POZ proteins include Tramtrack (Ttk) [28,29,31]; Mod(mdg4) [29,32]; Pipsqueak (Psq) [29,33] and Batman (Lolal) [29,34,35]. The GAF BTB/POZ domain has also been shown to contribute to interactions with non-BTB/POZ proteins, for example SAP18, a component of the Sin3-HDAC corepressor complex [36]. Finally, the alternative C-terminal domains could expand the range of possible GAF partners.

Despite the identification of a number of GAF partners, the scope of the GAF interacting protein network is unknown and its relationship to the diverse nuclear functions of the GAF protein remains poorly understood. In the studies reported here we have used a combination of immunoprecipitation and mass spectrometry to identify the proteins associated with GAF in nuclear extracts. Our data support the idea that the functional versatility of the GAF protein arises, at least in part, from its ability to associate with multiprotein complexes that have dedicated, but distinct functions in transcriptional regulation and chromosomal architecture. These complexes include the SWI/SNF and ISWI subfamilies of ATP-dependent chromatin remodelers, Polycomb (PcG) and Trithorax (TrxG) Group proteins, condensin, cohesion, and boundary/insulator-associated factors. Finally, several GAF network proteins were confirmed to interact directly with the GAF protein.

## Materials and methods

### Ethics statement

Animal handling for the antibody production was carried out strictly according to the procedures outlined in the NIH (USA) Guide for the Care and Use of Laboratory Animals. The protocols used were approved by the Committee on Bioethics of the Institute of Gene Biology, Russian Academy of Sciences. All procedures were performed under the supervision of a licensed veterinarian, under conditions that minimize pain and distress.

Rabbits were purchased from a licensed specialized nursery, Manihino. Soviet chinchilla rabbits used in the study are not endangered or protected. Only healthy rabbits, certified by a licensed veterinarian were used. The rabbits were individually housed in standard size, stainless steel rabbit cages and provided ad libitum access to alfalfa hay, commercial rabbit food pellets, and water. The appetite and behavior of each rabbit was monitored daily by a licensed veterinarian. Body weight and temperature of each rabbit were evaluated prior to and daily following immunization. No animals became ill or died at any time prior to the experimental endpoint. At the end of the study period all rabbits were euthanized by intravenous injection of barbiturate anesthetics.

### Antibodies

Antibodies against GAF (1–519 aa of isoform PC) [37], Iswi (1–96 aa of isoform PA), Bap55 (1–162 aa of isoform PA), Adf1 (full length of isoform PC), Su(z)12 (448–798 aa of isoform PA), E(z) (8–184 aa of isoform PA), eIF3-S8 (399–553 aa of isoform PA) were raised in rabbits.

Antigens for antibody production were expressed as a 6 × His-tagged fusion proteins in Escherichia coli, affinity purified on Ni Sepharose 6 Fast Flow (GE Healthcare) according to the manufacturer’s protocol and injected into rabbits following the standard immunization procedure. Antibodies were affinity-purified from serum on the same antigene as was used for immunization and tested by Western blotting and immunoprecipitation (IP) to confirm their specificity.

Other antibodies (used for the co-IP and EMSA experiments) were generously donated by Anton Golovnin (Mod(mdg4) common, Mod(mdg4)-PT (67.2) and Pzg/Z4), Elissa Lei (Mod(mdg4)-PT (67.2)), Oksana Maksimenko (E(y)2), Rakesh Mishra (Batman), Carl Wu (GAF), and David Gilmour (GAF).

### Isolation of GAGA factor-associated proteins and proteome sequencing

GAGA factor-associated proteins were isolated from the nuclear extracts of 0–12 hour *Drosophila* wild-type Oregon embryos (Bloomington Stock Center #5) prepared as described in [38,39] by immunoaffinity purification. The GAF antibody was coupled to the protein A Sepharose beads (Sigma) using DMP (Sigma) according to a published protocol [40] and loaded onto a column. Protein A sepharose with no antibody served as the negative control. The column was equilibrated with HEMG buffer (25 mM Hepes-KOH pH 7.6, 12.5 mM MgCl2, 0.1 mM EDTA, 10% glycerol, 1 mM DTT, and complete protease inhibitor mixture Roche) containing 150 mM NaCl (HEMG-150). Nuclear extract in the amount of 10 mg (by protein) was loaded on to the column containing the GAF antibody-protein A sepharose beads and incubated for two hrs. The column was washed extensively with HEMG-500 plus 0.1% Nonidet P-40 and then eluted with 0.1 M glycine at pH 2.5. Eluted proteins were analyzed mass spectrometry.

For analytical IP and co-IP the same procedure was followed. For a typical analytical experiment 100 μg of the nuclear extract was incubated with agarose A (mock control) or anti-GAF antibodies with or without DNaseI (0,5 U, Thermo Scientific). The co-IP’d proteins were detected by Western blotting.

### Mass spectrometry

In brief, proteins were reduced with 5 mM Tris(2-carboxyethyl)phosphine hydrochloride (Sigma-Aldrich, St. Louis, MO, product C4706) and alkylated with 2-chloroacetamide. Proteins were digested for 18 hr at 37°C in 2 M urea 100 mM Tris pH 8.5, 1 mM CaCl_2_ with 2 ug trypsin (Promega, Madison, WI, product V5111). Analysis was performed using an Agilent 1200 quaternary pump and a Thermo LTQ Orbitrap Velos using an in-house built electrospray stage [41]. Protein and peptide identification and protein quantitation were done with Integrated Proteomics Pipeline—IP2 (Integrated Proteomics Applications, Inc., San Diego, CA. http://www.integratedproteomics.com/). Tandem mass spectra were extracted from raw files using RawExtract 1.9.9 [42] and were searched against UniProt Drosophila melanogaster database with reversed sequences using ProLuCID [43,44]. The search space included all fully-tryptic and half-tryptic peptide candidates for the tryptic digest with static modification of 57.02146 on cysteine. Peptide candidates were filtered using DTASelect [42].

### EMSA experiments

Nuclear extracts from 6–18 hour *Drosophila* wild-type Oregon embryos, utilized for EMSAs were prepared using methods adopted from previously published procedures [45]. In the EMSAs, the binding reactions were performed in a 20 μL volume consisting of 25 mM Tris-Cl (pH 7.4), 100 mM KCl, 1 mM EDTA, 0.1 mM dithiothreitol, 0.1 mM PMSF, 0.03 mg/ml bovine serum albumin, 10% glycerol, 0.25 mg/ml poly(dA-dT)/poly(dA-dT) and 20 μg of protein derived from nuclear extract. The reaction mixtures containing the ^32^P labeled GAGA4 probe (see [46] for description of probe) were incubated for 30 minutes at room temperature with or without 20 μg of nuclear extracts (and plus antibodies as indicated) and loaded onto a pre-cleared 4% acrylamide:bis-acrylamide gels in 0.5 x TBE–2.5% glycerol gel. Binding reactions were electrophoresed at 180V for 3–4 hr with a 0.5 x TBE-2.5% glycerol running buffer at 4°C, dried and imaged using a Typhoon 9410 scanner and Image Gauge software and/or X-ray film.

### Two-hybrid analysis

Two-hybrid assays were carried out using yeast strain pJ69-4A, plasmids, and protocols obtained from Clontech. For growth assays, plasmids were transformed into pJ69-4A by the lithium acetate method as described by the manufacturer and were plated on nonselective media lacking tryptophan and leucine. After 3 days of growth at 30°C, plates were replicated on selective media: (1) lacking tryptophan, leucine, and histidine in the presence of 1mM or 5 mM 3-aminotriazole (3-AT); (2) lacking tryptophan, leucine, histidine and adenine. Each assay was repeated at least twice and growth was compared after 2, 4 and 7 days. Based on the extent of growth the interactions were scored as strong (+++), intermediate (++) or weak (+).

### Plasmids for Y2H assay

The full-length GAF isoform PC 1-519aa or GAF fragments 1–131 aa (BTB domain), 1–316 aa, 1–389 aa were cloned into pGBT9 vector (Clontech) to make fusions with the GAL4 DNA-binding domain. Other cDNA were cloned into pGAD24 vector to make fusion with the GAL4 activating domain. The BTB/POZ domain containing fragments were used for: Lola (CG12052) PA 1–120 aa; CG41099 PB 33–164 aa; Mri (CG1216) PA 86–210 aa; Bab2 (CG9102) PA 194–313 aa; CG8924 PB 1–146 aa. The full-length cDNAs were used in case of CP190 (CG6384 PA) 1–1096 aa; Batman (Lolal, CG5738 PA) 1-127aa; Sry-delta (CG17958 PA) 1–433 aa; L(3)neo38 (CG6930 PA) 1–380 aa; Pzg/Z4 (CG7752 PA) 1–996 aa; MEP-1 (CG1244 PA) 1–1152 aa; Su(Hw) (CG8573 PA) 1–941 aa; CG2199 PB 1–733 aa; Pita (CG3941 PA) 1–681 aa. The cDNA fragments were used for Row (CG8092 PA) 10–1281 aa; CG (CG8367) PF 31–467 aa.

## Results and discussion

We used immunoaffinity purification (IP) to identify proteins in nuclear extracts that are associated with GAF. Nuclear extracts were prepared from 0–12 hr wild type embryos (Figure A in S1 File) and incubated with a GAF polyclonal antibody that had been coupled to Protein A Sepharose beads. The polyclonal antibody was raised against the full-length 519 amino acid GAF isoform. After extensive washing the bound protein was eluted from the Protein A Sepharose beads with 0.1 M glycine at pH 2.5 [38] (IP, Figures B and C in S1 File) and analyzed by mass spectrometry [41,42]. These experiments were done in triplicate. Numerous proteins were detected in the samples isolated from the GAF antibody affinity beads that were absent in the sample prepared from the control beads (S2 File). Altogether proteins encoded by 2421 genes were present in at least one of GAF IP sample; however, slightly less than half (1202) were found in all 3 replicates (S3 File). Of those that were detected in all three experiments, only 903 had p-values <0.05 (S3 File). GO analysis of this latter group indicated that most of the proteins had biological functions likely to be relevant to GAF including chromosome organization, transcription, DNA replication, and mRNA processing and export (Figure D in S1 File). Also most (but not all) of the GAF associated proteins identified in previous studies were found in the immunoaffinity purified samples (S4 File). The different protein complexes and proteins that were found associated with GAF in nuclear extracts are discussed further below.

### GAF is associated with ATP-dependent chromatin remodelers

In agreement with previous studies [7,10], proteins derived from a collection of ATP-dependent chromatin remodeling complexes are found in the GAF IP samples. However, unlike these previous studies, we were able to detect most if not all of the known components of these complexes (Table 1, and see Table A in S5 File for detailed data). Moreover, with only a few exceptions, the protein components of these complexes had p-values <0.05. For the two SWI/SNF, PBAP and BAP [47], subfamily members we detected all of the (7) proteins that are common to both complexes. The co-purification of GAF with Bap55 was confirmed by co-IP using Bap55 specific antibodies (Figure E in S1 File). We also detected the three proteins, Polybromo, Bap170 and SAYP, that are specific to the PBAP complex, as well as the single BAP specific subunit, Osa [47,48]. The fact that all of the components of PBAP and BAP are found GAF associated would provide a compelling argument that GAF interacts with intact PBAP/BAP complexes, rather than with some type of sub-complex variants.

**Table 1:** GAF associated ATP-dependent chromatin remodelers.

Subunits	Annotation symbol	Peptide count			Physical connection to GAF	Subunits	Annotation symbol	Peptide count			Physical connection to GAF
		GAF IP	Mock	P-value				GAF IP	Mock	P-value	
GAF/Trl	CG33261	144|96|60	3|0|0	< 0.05		**ISWI subfamily**					
**SWI/SNF subfamily**											
**PBAP/BAP complex**						**NURF complex**					
Mor	CG18740	53|104|65	0|16|13	< 0.05	[7]	Iswi	CG8625	57|102|65	2|23|12	< 0.05	[7,10]
Brm	CG5942	33|63|45	0|11|5	< 0.05	[7]	E(bx)	CG32346	47|73|51	2|9|6	< 0.01	[7,10]
Bap111	CG7055	31|33|26	4|11|2	< 0.005		NURF-38	CG4634	27|34|23	5|3|3	< 0.01	
Bap55	CG6546	30|52|21	2|7|4	< 0.05	[7]	Caf1-55	CG4236	11|26|26	0|5|0	< 0.05	
Act5C	CG4027	23|83|56	4|25|16	= 0.071	[7]	**associated protein**					
Bap60	CG4303	23|47|26	5|3|6	< 0.05	[7]	Pzg/Z4	CG7752	48|47|36	0|9|4	< 0.001	[49]
Snr1	CG1064	21|19|8	0|0|3	< 0.05	[7]	**ACF complex**					
**PBAP specific subunits**						Iswi	CG8625	57|102|65	2|23|12	< 0.05	[7,10]
Polybromo	CG11375	51|80|54	0|11|4	< 0.01	[7]	Acf1	CG1966	39|74|34	0|13|9	< 0.05	
Bap170	CG3274	27|65|20	2|13|6	= 0.078		**ToRC complex**					
SAYP	CG12238	10|14|9	0|0|0	< 0.01		Iswi	CG8625	57|102|65	2|23|12	< 0.05	[7,10]
**BAP specific subunits**						CtBP	CG7583	22|30|22	14|3|0	< 0.05	
Osa	CG7467	17|40|26	0|12|6	< 0.05		Tou	CG10897	9|23|4	0|0|0	= 0.085	
**CHD subfamily**											
**dNURD (Mi-2) complex**											
Mi-2	CG8103	28|52|31	0|6|6	< 0.05	[7]	HDAC1	CG7471	11|35|15	0|3|0	= 0.059	[7]
MTA1-like	CG2244	11|24|11	0|3|2	< 0.05		Caf1-55	CG4236	11|26|26	0|5|0	< 0.05	
Simj/p66	CG32067	7|14|7	0|2|0	< 0.05		MBD-like	CG8208	3|5|3	0|2|0	< 0.05	
MEP-1	CG1244	6|15|14	0|3|0	< 0.05		CDK2AP1	CG18292	2|5|5	0|0|0	< 0.05	
**associated protein**											
Ttk	CG1856	22|23|8	0|0|0	< 0.05	[28,29,31]						

Components of ATP-dependent chromatin complexes that were detected in the GAF IP samples from 0–12 h embryonic nuclear extracts are listed. The peptide counts for each of the three GAF IPs or control Mock probes are presented. In the right column we’ve listed the references for proteins previously identified as physically associated with GAF. Statistical significance was analyzed using the Student’s t-test and expressed as a P-value.

The same is true for the ISWI subfamily complexes NURF, ACF and ToRC, and the CHD subfamily complex dNURD. There are four known NURF complex proteins, Iswi, E(bx)/NURF301, NURF-38 and Caf1-55/p55 [9,50], plus an associated protein Pzg/Z4 [51]. All five of these proteins are detected in the GAF immunoaffinity sample (Table 1, and Table A in S5 File). Similarly, known subunits of the ACF complex [52], Iswi and Acf1, and ToRC complex [53], Iswi and CtBP and Tou, are present. The CHD family remodeler, dNURD, has eight subunits, Mi-2, MEP-1, MTA1-like, Simj/p66, HDAC1, Caf1-55, MBD-like, CDK2AP1 [54] and Ttk as associated protein [54,55]. All of these proteins are immunoaffinity purified from nuclear extracts with the GAF antibody. The association of GAF with Iswi (NURF, ACF) and Pzg/Z4 was additionally confirmed by co-IP (Figure E in S1 File).

While these findings provide strong evidence that GAF associates with multiple remodeling complexes, these interactions need not be direct and could be mediated by accessorial proteins. For example, like GAF, Ttk has an N-terminal BTB/POZ domain and a C-terminal zinc finger DNA binding domain. The Ttk BTB/POZ is known to interact directly with the dNURD subunits, MEP-1 and Mi-2 [54,55]. It also interacts directly with the GAF BTB/POZ domain [28,29,31]. The same is true for GAF interactions with the NURF complex. Previous studies have shown that the zinc finger protein, Pzg/Z4 interacts not only with dNURF [51], but also with GAF [49]. Moreover, as described further below, this GAF:Pzg/Z4 interaction is direct. Thus, GAF association with both the dNURD and NURF remodeling complexes could be mediated *in vivo* by intermediary zinc finger DNA binding proteins, rather than by GAF itself.

### Components of the transcription machinery

In addition to its role in the formation and maintenance of nucleosome free regions over promoters, GAF protein has been implicated at other steps in the transcription process, including recruitment of transcription factors and Pol II to promoters, promoter pausing and mRNA biogenesis. In S6 File we have listed the proteins that are subunits of factors that function in transcription and/or mRNA biogenesis. While subunits from RNA Pol II, Mediator, TFIID and NELF can be detected in the GAF immunoaffinity sample, the number of peptides and the spectral counts for the proteins in these complexes are typically rather low and not reproduced well in biological replicates. Moreover, unlike the chromatin remodeling factors, proteins known to be components of these complexes (e.g, Rbp4 and Rbp12 for RNA Pol II) aren’t detected. Given that these factors frequently co-localize with GAF at promoters or just downstream, it seems likely that GAF association either reflects their co-localization on the DNA or is not especially robust.

The TREX-THO complex functions in transcription elongation, mRNA biogenesis and mRNA export [56–59]. Supporting the findings reported in a previous study [7] we detected all of the subunits of TREX–THO complex (Tho2 and Hpr1) in all GAF immunoaffinity samples (S6 File). GAF was also shown to associate with the FACT complex proteins Ssrp and Dre4 [7,8]. FACT acts subsequent to transcription initiation to release RNA polymerase II from a nucleosome-induced block [60]. Only one of the two FACT complex proteins, Ssrp was detected in the GAF immunoaffinity samples, and both the peptide and spectral counts were quite low (S6 File). Thus, at least under our conditions, FACT does not seem to be stably associated with GAF.

### GAF associated PcG/TrxG factors

Polycomb-dependent silencing in *Drosophila* is typically mediated by special cis-acting elements called Polycomb Response Elements (PRE). These elements recruit the PRC1, PRC2 and PhoRC complexes, which then function to silence genes in the neighborhood [61–65]. Like promoters and other regulatory elements, PREs are nucleosome free and have recognition sites for DNA binding proteins such as Pho, Cg, Zeste, Psq, Adf1, Grh, Dsp1 and Spps, which have been implicated in PcG silencing [63,66,67]. Like these DNA binding proteins, GAF also binds to PRE sequences *in vitro* and *in vivo* and has been shown to be important for their PcG dependent silencing activity [16–18,68]. Several additional non-DNA binding PcG proteins that are required for homeotic gene silencing but have not been assigned to PcG complexes have also been described, including Sxc/Ogt [69], Dom [70], and Batman (Lolal) [34].

Of these factors, we find that the Adf1 and Zeste DNA-binding proteins are present in all IP samples with p-values of < 0.01 reflecting a stable association with GAF. The presence of Adf1 was confirmed by Western blotting (Figure E in S1 File). With respect to the three major Polycomb group complexes, all of the PRC2 subunits are present in all three GAF immunoaffinity purifications and have p-values of <0.05 (Table 2, and Table B in S5 File). To further verify GAF- PRC2 association, we probed for the E(z) and Su(z)12 subunits of the PRC2 complex in GAF IPs using Western blotting (Figure E in S1 File).

**Table 2:** GAF associated TrxG/PcG factors.

Subunits	Annotation symbol	Peptide count			Physical connection to GAF	Subunits	Annotation symbol	Peptide count			Physical connection to GAF
		GAF IP	Mock	P-value				GAF IP	Mock	P-value	
GAF/Trl	CG33261	144|96|60	3|0|0	< 0.05							
**Polycomb Group Proteins**											
**PRC2 complex**											
Su(z)12	CG8013	15|13|4	0|0|0	< 0.05		E(z)	CG6502	10|13|4	0|0|0	< 0.05	
Caf1-55	CG4236	11|26|26	0|5|0	< 0.05		Esc	CG14941	7|9|3	0|2|0	< 0.05	[71]
**PhoRC complex**											
Sfmbt	CG16975	3|5|3	0|0|0	< 0.05							
Pho	CG17743	5|0|3	0|0|0	= 0.104							
**PRC1 complex**											
Sce/dRing	CG5595	5|7|3	0|0|0	< 0.05		Ph-d	CG3895	3|0|0	0|0|0	= 0.211	
Ph-p	CG18412	4|0|0	0|0|0	= 0.211	[35]	Pc	CG32443	**0|0|0**	0|2|0	ND	[71]
Psc	CG3886	3|4|0	0|2|0	= 0.154							
**PRC1 associated proteins**											
l(1)G0020	CG1994	44|40|14	5|3|0	< 0.05		Ppl-87B	CG5650	19|37|18	3|2|2	< 0.05	
26-29-p	CG8947	26|33|22	2|6|4	< 0.01		P90	CG10077	19|38|20	2|4|3	< 0.05	
**Other Proteins associated with Polycomb or with Polycomb/Trithorax Function**											
**DNA-binding factors**											
Adf1	CG15845	9|11|7	0|3|0	< 0.005		Zeste	CG7803	3|3|2	0|0|0	< 0.01	
Psq/BTB-V	CG2368	12|4|3	0|0|0	= 0.078	[29,33,49]	Combgap	CG8367	7|8|3	0|4|2	= 0.055	
**Non-DNA-binding factors**											
Sxc/Ogt	CG10392	17|18|14	0|0|0	< 0.005		Dom	CG9696	13|20|14	0|6|0	< 0.01	
Batman/Lolal	CG5738	19|18|11	2|3|3	< 0.05	[29,34,35]	Kto	CG8491	8|14|4	0|0|0	< 0.05	
Hcf	CG1710	54|68|41	0|17|15	< 0.01							
**Proteins associated with Trithorax Function**											
**DNA-binding factors**											
Fs(1)h	CG2252	8|5|8	0|0|0	< 0.01	[72]						
**Non-DNA-binding factors**											
Lid	CG9088	49|71|40	0|4|0	< 0.05		Vtd/Rad21	CG17436	7|10|5	0|0|0	< 0.05	
Ash2	CG6677	17|23|11	0|0|0	< 0.05		Kis	CG3696	11|23|13	0|3|0	< 0.05	[7]
Brel	CG10542	15|7|9	0|0|0	< 0.05							

This table lists GAF associated PcG and TrxG protein present in GAF IP samples. Proteins that are thought to be functionally relevant to PcG and/or TrxG activity PcG group are also included in this Table. Other designations are as in Table 1.

Also present in the GAF immunoaffinity sample are components of the PhoRC and PRC1 complexes. However, the peptide and spectral counts for PhoRC and PRC1 proteins are quite low and most of the PRC1 components are not present in all three immunoaffinity purifications. Additionally, Pc isn’t even detected (Table 2, and Table B in S5 File). These findings would be consistent with the idea that GAF—PRC1/PhoRC association is not especially stable and is likely to be indirect either via other proteins (for example, Batman or by interactions with accessory factors such as the putative acetyltransferase l(1)G0020 or the helicase p90. It is also worth noting that in the case of PRC2, which seems to be GAF associated, the peptide counts are less than that observed for, for example, components of the PBAP/BAP complexes (Table 1, and Table A in S5 File).

We also detected several known TrxG factors in the GAF immunoaffinity purified sample. Besides members of BAP/PBAP complexes (Brm, Mor, Snr1, Osa, SAYP) [48,73–75] listed in Table 1, these include: Fs(1)h [76,77], Ash2 [77], Vtd, Kis [75], Lid [78], Bre1 [79] (Table 2).

### GAGA-associated factors implicated in boundary function

GAF-binding sites were shown to be required for the insulator activity of chromatin domain boundary elements [80,81] and the GAF protein localizes to many known or putative insulators *in vivo* [22]. One of the boundary elements that requires GAF for its insulator activity is the *Fab-7* boundary from the *Drosophila* Bithorax complex [81,82]. There are six GAGA motifs in the major *Fab-7* nucleosome free region (nuclease hypersensitive site HS1). Four of these are located in a 293 bp long fragment called dHS1 that has developmentally restricted insulator activity in transgene assays, functioning (primarily) from mid-embryogenesis through to the adult stage. Recent studies have shown that a large ~700 kD complex named LBC binds to probes containing three of the four GAGA motifs in dHS1 [46]. In each case, the minimal recognition sequence for the LBC is more than 65 bp. Supershift experiments with antibodies directed against known insulator proteins together with gel filtration experiments indicate that the LBC complex contains GAF, Mod(mdg4) and E(y)2. Thus unlike the other complexes that interact with GAF (e.g., PABP/BAP or NURF remodeling complexes) either directly or indirectly, GAF is an integral component of the LBC. As would be expected if the LBC were pulled down by the GAF antibody, both Mod(mdg4) and E(y)2 are present in the affinity purified sample (Table 3, and Table C in S5 File).

**Table 3:** GAF associated chromosome architecture factors.

Subunits	Annotation symbol	Peptide count			Physical connection to GAF	Subunits	Annotation symbol	Peptide count			Physical connection to GAF
		GAF IP	Mock	P-value				GAF IP	Mock	P-value	
**LBC complex**						**Cohesin complex**					
GAF/Trl	CG33261	144|96|60	3|0|0	< 0.05		SMC3	CG9802	51|65|35	0|6|3	< 0.05	
Mod(mdg4)	CG32491	14|16|12	0|9|0	< 0.05	[29,32,83]	SMC1	CG6057	44|53|34	0|8|2	< 0.005	
E(y)2/ ENY2	CG15191	6|4|3	0|2|2	< 0.05		SA/SCC3	CG3423	22|43|13	0|0|0	< 0.05	
**CP190 complex**						Vtd/Rad21	CG17436	7|10|5	0|0|0	< 0.05	
CP190	CG6384	23|12|9	0|7|2	< 0.05		**associated proteins**					
Su(Hw)	CG8573	11|19|12	0|0|0	< 0.05		Pds5	CG17509	21|34|18	0|2|0	< 0.05	
Mod(mdg4)	CG32491	14|16|12	0|9|0	< 0.05	[29,32,83]	Nipped-B	CG17704	9|19|10	0|4|0	< 0.05	
Map60/CP60	CG1825	14|25|14	0|0|3	< 0.05		**Condensin I**					
Pita	CG3941	6|9|2	0|2|0	= 0.06		SMC2	CG10212	47|51|42	0|4|6	< 0.001	
**associated proteins**						Glu	CG11397	43|77|55	0|14|6	< 0.05	
Pzg/Z4	CG7752	48|47|36	0|9|4	< 0.001	[49]	Barr	CG10726	18|19|17	0|0|2	< 0.001	
Dref	CG5838	60|82|28	7|14|9	< 0.05		Cap-G	CG34438	15|26|5	0|0|0	= 0.064	
Chro	CG10712	32|38|20	0|10|6	< 0.05		Cap-D2	CG1911	13|32|10	0|2|2	= 0.065	

Known members of the LBC complex and the CP190-associated proteins are listed. Other designations are as in Table 1.

As noted in the introduction, previous studies have shown that the N-terminal BTB/POZ domains of the GAF and Mod(mdg4) mediates both self-interactions and interactions between these two proteins. The GAF BTB/POZ domain tends to form homodimers, while the Mod(mdg4) BTB/POZ domain forms octamers. Interestingly, there are 31 predicted isoforms of Mod(mdg4). All share the N-terminal BTB/POZ protein interaction domain, but have unique C-terminal FLYWCH zinc finger DNA binding domains. Since GAF-Mod(mdg4) interactions are mediated by the BTB/POZ domains, the LBC could contain a complex mixture of Mod(mdg4) isoforms. Consistent with this possibility, we detect a total of 14 Mod(mdg4) isoforms in the GAF IP samples (S7 File). As we don’t know the relative abundance of the different Mod(mdg4) isoforms in 0–12 hr embryonic nuclear extracts, we can’t exclude the possibility that there are yet other GAF-associated Mod(mdg4) isoforms in embryos. Also while we cannot be certain that all of the isoforms detected are derived from LBC complexes, it seems likely that this is the case as they all have the Mod(mdg4) BTB/POZ domain that is responsible for interactions with GAF.

**Fig 1:**
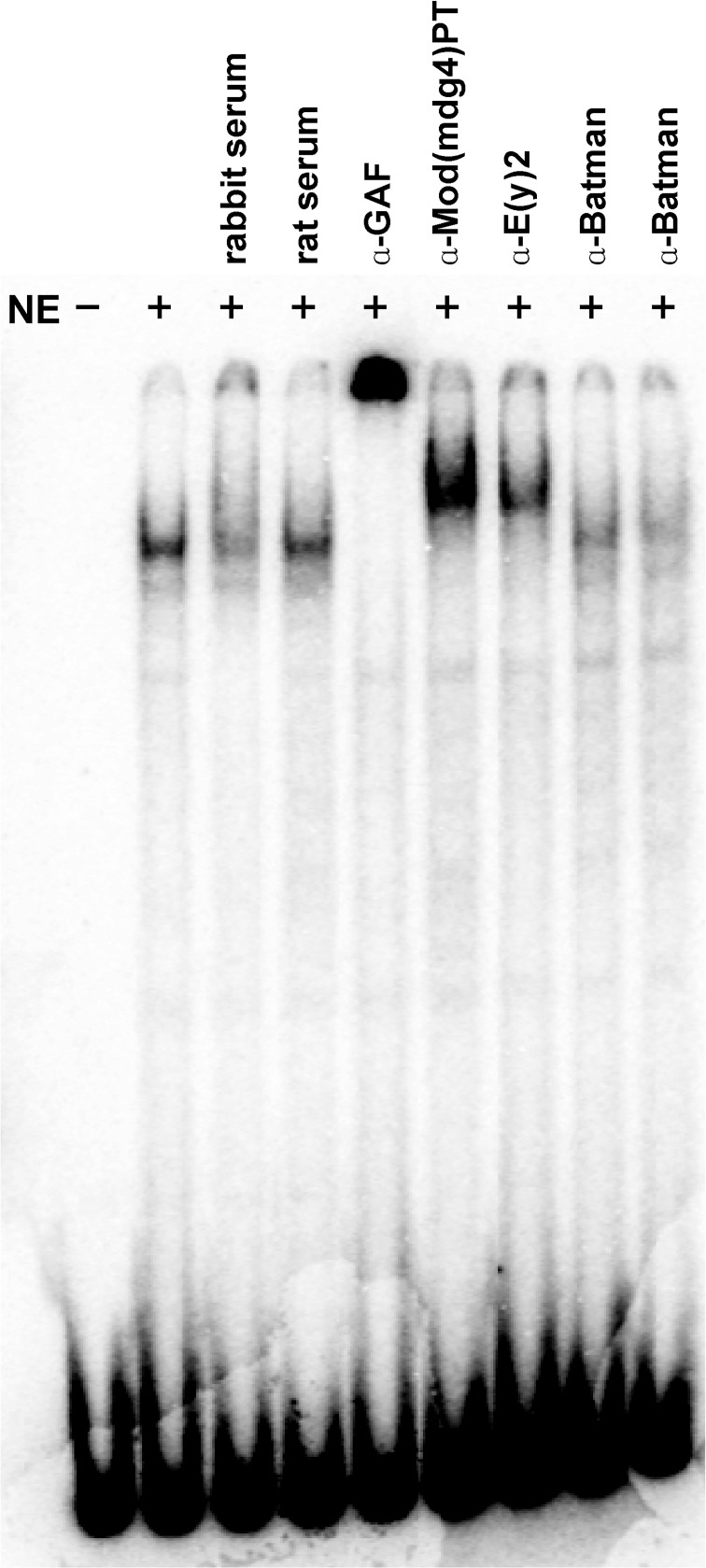
The LBC contains the Mod(mdg4) PT (67.2) isoform. The GAGA4 probe [46] from the *Fab-7* sub-element dHS1 was incubated with “late” 6–18 hr embryonic nuclear extracts (NE). Included in the incubation mixture were control rabbit and rat serum or polyclonal antibodies direct against GAF, E(y)2, the PT Mod(mdg4) isoform, or Batman as indicated. The samples were then analyzed by gel electrophoresis as described in the materials and methods.

One of the Mod(mdg4) isoforms in the GAF IP is PT [(Mod(mdg4)67.2 or 2.2] which has been implicated in *su(Hw)* dependent insulator function [84,85]. We first confirmed that this specific isoform is present in GAF IP by co-IP (Figure E in S1 File). At the next step, to confirm that Mod(mdg4)PT is present in the LBC we did EMSA experiments with a probe, GAGA4, that spans one of the dHS1 GAGA motifs (#4). As shown in Fig 1, the GAGA4 probe generates a characteristic slowly migrating shift when incubated with embryonic nuclear extracts. The yield of this shift is slightly reduced when control rabbit serum is included in the reaction mix, while it is not greatly affected by control rat serum. As previously described, LBC supershifts are observed when antibodies against GAF and E(y)2 are included in the reaction mix. In contrast, antibodies against another BTB/POZ domain protein, Batman, which physically interacts with GAF *in vitro* and *in vivo* ([29,34,35], see below) don’t generate supershifts. This finding would argue that the LBC probably doesn’t contain Batman and that there must be some other, as yet unidentified, GAF:Batman complex. Importantly, antibodies specific for the PT isoform of Mod(mdg4) generate a supershift, indicating that this isoform is present in LBC complexes.

In addition to the LBC, GAF appears to be associated with other factors known to be involved in boundary activity. These include Pzg/Z4, CP190, and Su(Hw) and the boundary associated factors Dref and Chro (Table 3, and Table C in S5 File). Moreover, key subunits of the chromosome architectural complexes, cohesin and condensin are also present in the GAF immunoaffinity purified sample (Table 3, and Table C in S5 File).

### Testing for direct interactions with GAF

Though many proteins are found associated with GAF after immunoaffinity purification, only a subset are expected to interact directly with GAF. Instead, many will be present in the purified sample because they linked to GAF through common interactions with intermediary proteins. To begin classifying the proteins in the GAF immunoaffinity sample as either direct or indirect interactors, we took advantage of the yeast two-hybrid assay.

#### BTB/POZ domain GAF partners

Previous studies have shown that the BTB/POZ domain of GAF interacts with several other BTB/POZ domain proteins. Thus we expected to find GAF associated BTB/POZ domain proteins. Altogether 11 different BTB/POZ proteins were present in all 3 GAF IP samples (S8 File). These includes four previously identified direct partners of GAF: Ttk [28,29,31], Batman [29,34,35], Psq/BTB-V [29,33,49], and Mod(mdg4) [29,32,83]. The BTB-domains of these four proteins have been shown to interact with the BTB-domain of GAF [28,29,31–33]. Due a high degree of sequence similarity these and two other proteins found in all GAF immunoaffinity samples, Lola/BTB-IV and Bab2/BTB-II, have been classified as Ttk Group BTB/POZ domain proteins [29,86]. In addition, there are two other proteins, CG8924 and Rib, in all GAF immunoaffinity samples that have BTB/POZ domains which display a high degree of sequence similarity to members of the Ttk group. Finally, there are three GAF associated proteins, CP190, CG41099, Mri, that have a more distantly related BTB/POZ domain (S9 File).

**Fig 2:**
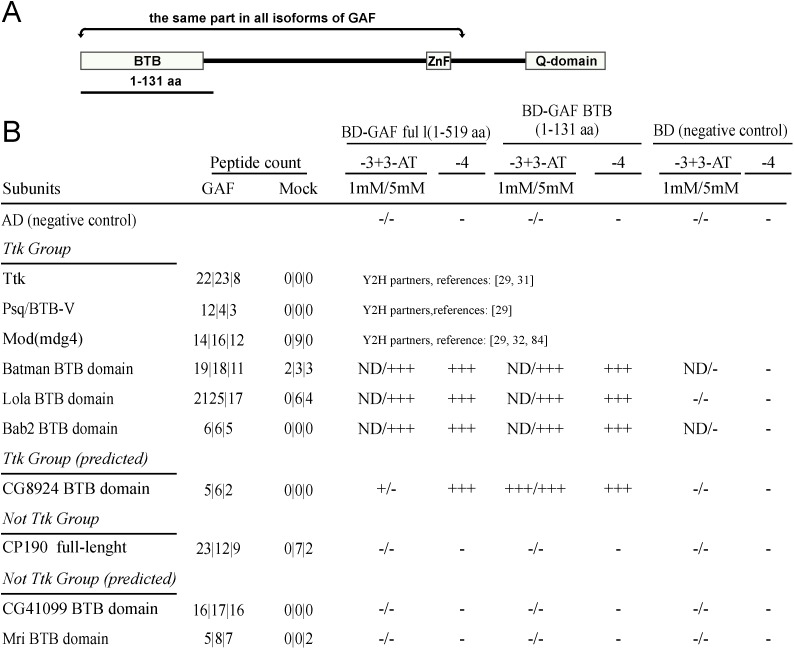
Y2H analysis of GAF direct BTB/POZ domain partners. (A) The structure of GAF protein. The N-terminal BTB/POZ, a central C2H2-type zinc finger and several alternative glutamine rich (Q) C-terminal domains are indicated. The 1–131 aa GAF was used in Y2H analysis as BTB/POZ containing fragment. (B) The test of ability of the BTB/POZ proteins to interact with GAF in yeast two-hybrid assay. The data on the left lists the peptide count of tested proteins in the GAF immunoaffinity purified samples. The two-hybrid assay was performed with full length 519 aa GAF isoform or with BTB/POZ GAF containing fragment (1–131 aa indicated in the scheme above) fused to GAL4 binding domain (BD). The BTB/POZ domain of each protein in this group was fused with GAL4 activation domain (AD). Three types of selective media was used: lacking tryptophan, leucine, and histidine in the presence of 1mM or 5 mM 3-aminotriazole (3-AT) (-3+3-AT) and lacking tryptophan, leucine, histidine and adenine (-4). The +++ (strong), ++ (middle), + (weak), indicates the extent of growth that was detected at day 2, day 4 or day 7 respectively.

For the yeast two-hybrid experiments, we fused the GAL4 DNA binding domain to full length GAF 519 aa isoform or to an N-terminal GAF fragment 1–131 aa that contains the BTB/POZ domain. To test for interactions, we then fused the BTB/POZ domains of the GAF associated BTB/POZ proteins to the GAL4 activation domain. Since direct interactions have already been demonstrated for four of these BTB/POZ domain proteins (see above and Fig 2), we selected only one, Batman, as a positive control. The full-length CP190 protein that previously failed interact with GAF [29] was used as a negative control.

As was found previously for a subset of the Ttk-group BTB/POZ members [29], all of the new proteins in this group interact directly with GAF through BTB/POZ domains (Fig 2). Most of these proteins have several different isoforms, all of which share their BTB/POZ domain. This means that the complexes containing GAF and these Trk-group proteins band could contain different isoform combinations. The other BTB/POZ protein besides Mod(mdg4) that has an extraordinarily large number of isoforms is Lola, which has around 20 distinct isoforms. Like Mod(mdg4) (see above) most of these isoforms have a unique C-terminal DNA binding domain. Altogether we were able to identify four different Lola isoforms in GAF immunoaffinity purified samples (S10 File).

While all members of the Ttk-group can interact directly with GAF, this is not true for the two other BTB/POZ domain proteins found associated with GAF. As indicated in Fig 2, we were unable to detect direct interactions between the BTB/POZ domains of CG41099, Mri proteins and GAF using the yeast two hybrid assay.

#### C2H2-type Zinc finger domain partners

**Fig 3:**
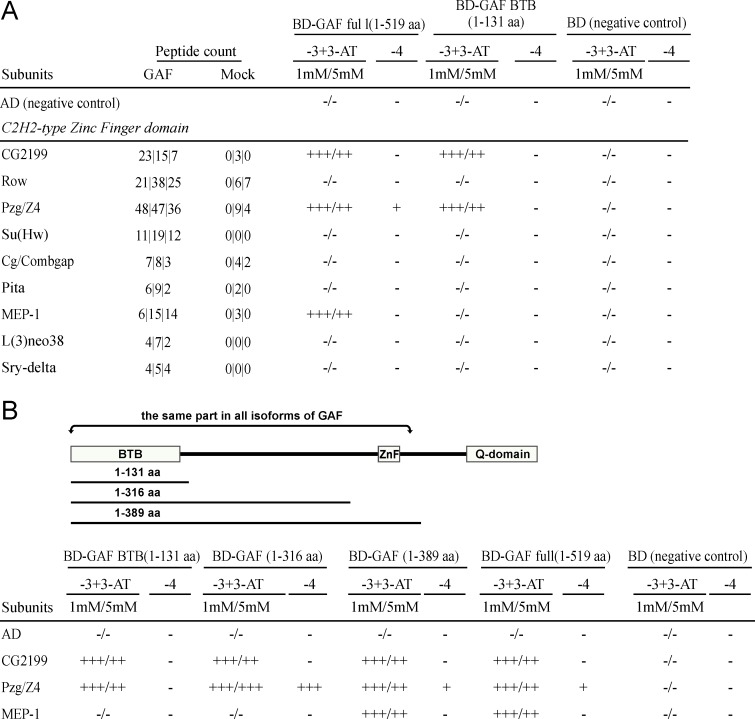
Y2H analysis of GAF direct C2H2-type Zinc Finger domain partners. (A) The test of ability of proteins with C2H2-type Zinc Finger domains to interact with GAF in the yeast two-hybrid assay. (B) Identification of the GAF domain that mediates interactions with the 3 interacting Zinc Finger proteins. The GAF deletion variants tested are indicated: 1–131 aa BTB GAF domain; 1–316 aa and 1–389 aa GAF fragments lacking or including the C2H2-type ZnF domain. Other designations are as in Fig 2.

Another class of proteins that could potentially interact directly with GAF is C2H2-type Zinc finger domain proteins. Like GAF, these proteins are also expected to bind directly to DNA. Altogether a total of 26 GAF associated proteins that have C2H2-type Zinc fingers were present in all three of the GAF immunoaffinity samples (S8 File). As discussed above, a few of these resemble GAF in that they also have a BTB/POZ domain (Lola/BTB-IV, Ttk, CP190). To extend our analysis, we selected 9 of non-BTB/POZ domain proteins to test for direct interactions with GAF (see Fig 3). Included in this list are the boundary proteins, Su(Hw), Pita [87,88], and two proteins that have been shown to associate with GAF in extracts, but not tested for direct interaction, Pzg/Z4 and CG2199 [49].

For these experiments we fused cDNAs encoding these proteins in frame to the GAL4 activation domain. Of the 9 proteins that we tested, only three (Pzg/Z4, CG2199 and MEP-1) were able to interact directly with full-length GAF in our two-hybrid assay (Fig 3). Since previous studies have shown that the BTB/POZ domain of GAF mediates its interactions with other BTB/POZ proteins, we wondered whether this domain is also responsible for interactions with proteins that lack a BTB/POZ domain. To address this question we generated a series of GAF truncations and then tested whether they retained the ability to interact with these three C2H2-type Zinc finger domain proteins. We found that two of the proteins, Pzg/Z4 and CG2199, appear to interact with GAF via its BTB/POZ domain (Fig 3). However, the MEP-1 requires a small region of the GAF protein that spans the single C2H2-type Zinc finger domain (Fig 3). This finding raises the possibility that the GAF Zinc finger domain not only has DNA binding activity but can also participate in protein-protein interactions.

## Conclusions

The GAF protein has extraordinarily diverse array of functions in *Drosophila*. Amongst the many functions that have so far been identified are generating nucleosome free regions of chromatin, transcriptional activation and elongation, PcG and heterochromatic silencing, chromatin domain boundaries and mitosis. The studies reported here provide a plausible explanation for how GAF can have so many different, and in some instances seemingly contradictory functions. Using immunoaffinity purification combined with mass spectrometry we have isolated proteins that are associated with GAF in nuclear extracts prepared from 0–12 hr embryos. For many of known GAF functions we have identified GAF-associated proteins and/or multi-subunit complexes that have the appropriate activities. For example, we detect both of SWI/SNF chromatin remodeling complexes, the CHD family complex, and the ISWI family complexes NURF, ACF and ToRC. Moreover, in contrast to previous studies all of the known subunits for these complexes are present in the GAF immunoaffinity sample, providing compelling evidence that GAF interacts with fully functional remodeling complexes. The same seems to be true for TREX-THO, cohesion and condensin I complexes. For some of the GAF associated proteins we have shown that interactions with GAF are direct. Finally, it is likely that the GAF associated proteins we have identified is still an incomplete list. There are a number of reasons for this. One is that not all complexes will remain intact during the affinity purification. Another limitation is that of antibody accessibility. Even though we used polyclonal antibodies, there may be complexes in which key GAF epitopes are not readily accessible, thus substantially reducing the yield of these protein/complexes in the immuonaffinity purified sample.

## Supporting information

S1 File Supplementary figures A-E.
(PDF)

S2 File GAF associated proteins. (XLSX)

S3 File GAF associated gene products. (XLSX)

S4 File Previously identified GAF partners. (XLSX)

S5 File GAF IP Tables A-C.
(XLS)

S6 File GAF IP Selected Factors 2. (XLSX)

S7 File Mod(mdg4) isoforms in GAF IP. (XLSX)

S8 File BTB and C2H2-type Zinc Finger proteins in GAF IP. (XLSX)

S9 File BTB proteins alignment. (PDF)

S10 File Lola isoforms in GAF IP. (XLSX)

## References

[1] Biggin MD, Tjian R (1988) Transcription factors that activate the Ultrabithorax promoter in developmentally staged extracts. Cell 53: 699–711. 2897243 10.1016/0092-8674(88)90088-8

[2] Soeller WC, Oh CE, Kornberg TB (1993) Isolation of cDNAs encoding the Drosophila GAGA transcription factor. Mol Cell Biol 13: 7961–7970. 7504178 10.1128/mcb.13.12.7961-7970.1993 PMC364868

[3] Soeller WC, Poole SJ, Kornberg T (1988) In vitro transcription of the Drosophila engrailed gene. Genes Dev 2: 68–81. 3356339 10.1101/gad.2.1.68

[4] Farkas G, Gausz J, Galloni M, Reuter G, Gyurkovics H, Karch F. (1994) The Trithorax-like gene encodes the Drosophila GAGA factor. Nature 371: 806–808. 10.1038/371806a0 7935842

[5] Tsukiyama T, Becker PB, Wu C (1994) ATP-dependent nucleosome disruption at a heat-shock promoter mediated by binding of GAGA transcription factor. Nature 367: 525–532. 10.1038/367525a0 8107823

[6] Okada M, Hirose S (1998) Chromatin remodeling mediated by Drosophila GAGA factor and ISWI activates fushi tarazu gene transcription in vitro. Mol Cell Biol 18: 2455–2461. 9566866 10.1128/mcb.18.5.2455 PMC110625

[7] Nakayama T, Shimojima T, Hirose S (2012) The PBAP remodeling complex is required for histone H3.3 replacement at chromatin boundaries and for boundary functions. Development 139: 4582–4590. 10.1242/dev.083246 23136390

[8] Shimojima T, Okada M, Nakayama T, Ueda H, Okawa K, Iwamatsu A, et al (2003) Drosophila FACT contributes to Hox gene expression through physical and functional interactions with GAGA factor. Genes Dev 17: 1605–1616. 10.1101/gad.1086803 12815073 PMC196133

[9] Tsukiyama T, Wu C (1995) Purification and properties of an ATP-dependent nucleosome remodeling factor. Cell 83: 1011–1020. 8521501 10.1016/0092-8674(95)90216-3

[10] Xiao H, Sandaltzopoulos R, Wang HM, Hamiche A, Ranallo R, Lee KM, et al (2001) Dual functions of largest NURF subunit NURF301 in nucleosome sliding and transcription factor interactions. Mol Cell 8: 531–543. 11583616 10.1016/s1097-2765(01)00345-8

[11] Leibovitch BA, Lu Q, Benjamin LR, Liu Y, Gilmour DS, Elgin SC. (2002) GAGA factor and the TFIID complex collaborate in generating an open chromatin structure at the Drosophila melanogaster hsp26 promoter. Mol Cell Biol 22: 6148–6157. 10.1128/MCB.22.17.6148-6157.2002 12167709 PMC134011

[12] Weber JA, Taxman DJ, Lu Q, Gilmour DS (1997) Molecular architecture of the hsp70 promoter after deletion of the TATA box or the upstream regulation region. Mol Cell Biol 17: 3799–3808. 9199313 10.1128/mcb.17.7.3799 PMC232231

[13] Fuda NJ, Guertin MJ, Sharma S, Danko CG, Martins AL, Siepel A, et al (2015) GAGA factor maintains nucleosome-free regions and has a role in RNA polymerase II recruitment to promoters. PLoS Genet 11: e1005108 10.1371/journal.pgen.1005108 25815464 PMC4376892

[14] Lee C, Li X, Hechmer A, Eisen M, Biggin MD, Venters BJ, et al (2008) NELF and GAGA factor are linked to promoter-proximal pausing at many genes in Drosophila. Mol Cell Biol 28: 3290–3300. 10.1128/MCB.02224-07 18332113 PMC2423147

[15] Li J, Liu Y, Rhee HS, Ghosh SK, Bai L, Pugh BF, et al (2013) Kinetic competition between elongation rate and binding of NELF controls promoter-proximal pausing. Mol Cell 50: 711–722. 10.1016/j.molcel.2013.05.016 23746353 PMC3695833

[16] Hagstrom K, Muller M, Schedl P (1997) A Polycomb and GAGA dependent silencer adjoins the Fab-7 boundary in the Drosophila bithorax complex. Genetics 146: 1365–1380. 9258680 10.1093/genetics/146.4.1365 PMC1208081

[17] Horard B, Tatout C, Poux S, Pirrotta V (2000) Structure of a polycomb response element and in vitro binding of polycomb group complexes containing GAGA factor. Mol Cell Biol 20: 3187–3197. 10757803 10.1128/mcb.20.9.3187-3197.2000 PMC85613

[18] Mishra RK, Mihaly J, Barges S, Spierer A, Karch F, Hagstrom K, et al (2001) The iab-7 polycomb response element maps to a nucleosome-free region of chromatin and requires both GAGA and pleiohomeotic for silencing activity. Mol Cell Biol 21: 1311–1318. 10.1128/MCB.21.4.1311-1318.2001 11158316 PMC99583

[19] Bhat KM, Farkas G, Karch F, Gyurkovics H, Gausz J, Schedl P. (1996) The GAGA factor is required in the early Drosophila embryo not only for transcriptional regulation but also for nuclear division. Development 122: 1113–1124. 8620838 10.1242/dev.122.4.1113

[20] Ohtsuki S, Levine M (1998) GAGA mediates the enhancer blocking activity of the eve promoter in the Drosophila embryo. Genes Dev 12: 3325–3330. 9808619 10.1101/gad.12.21.3325 PMC317233

[21] Kvon EZ, Stampfel G, Yanez-Cuna JO, Dickson BJ, Stark A (2012) HOT regions function as patterned developmental enhancers and have a distinct cis-regulatory signature. Genes Dev 26: 908–913. 10.1101/gad.188052.112 22499593 PMC3347788

[22] Negre N, Brown CD, Shah PK, Kheradpour P, Morrison CA, Henikoff JG, et al (2010) A comprehensive map of insulator elements for the Drosophila genome. PLoS Genet 6: e1000814 10.1371/journal.pgen.1000814 20084099 PMC2797089

[23] Negre N, Hennetin J, Sun LV, Lavrov S, Bellis M, White KP, et al (2006) Chromosomal distribution of PcG proteins during Drosophila development. PLoS Biol 4: e170 10.1371/journal.pbio.0040170 16613483 PMC1440717

[24] Schuettengruber B, Ganapathi M, Leblanc B, Portoso M, Jaschek R, Tolhuis B, et al (2009) Functional anatomy of polycomb and trithorax chromatin landscapes in Drosophila embryos. PLoS Biol 7: e13 10.1371/journal.pbio.1000013 19143474 PMC2621266

[25] van Steensel B, Delrow J, Bussemaker HJ (2003) Genomewide analysis of Drosophila GAGA factor target genes reveals context-dependent DNA binding. Proc Natl Acad Sci U S A 100: 2580–2585. 10.1073/pnas.0438000100 12601174 PMC151383

[26] Yanez-Cuna JO, Arnold CD, Stampfel G, Boryn LM, Gerlach D, Rath M, et al (2014) Dissection of thousands of cell type-specific enhancers identifies dinucleotide repeat motifs as general enhancer features. Genome Res 24: 1147–1156. 10.1101/gr.169243.113 24714811 PMC4079970

[27] Pedone PV, Ghirlando R, Clore GM, Gronenborn AM, Felsenfeld G, Omichinski JG. (1996) The single Cys2-His2 zinc finger domain of the GAGA protein flanked by basic residues is sufficient for high-affinity specific DNA binding. Proc Natl Acad Sci U S A 93: 2822–2826. 8610125 10.1073/pnas.93.7.2822 PMC39717

[28] Bardwell VJ, Treisman R (1994) The POZ domain: a conserved protein-protein interaction motif. Genes Dev 8: 1664–1677. 7958847 10.1101/gad.8.14.1664

[29] Bonchuk A, Denisov S, Georgiev P, Maksimenko O (2011) Drosophila BTB/POZ domains of "ttk group" can form multimers and selectively interact with each other. J Mol Biol 412: 423–436. 10.1016/j.jmb.2011.07.052 21821048

[30] Katsani KR, Hajibagheri MA, Verrijzer CP (1999) Co-operative DNA binding by GAGA transcription factor requires the conserved BTB/POZ domain and reorganizes promoter topology. EMBO J 18: 698–708. 10.1093/emboj/18.3.698 9927429 PMC1171162

[31] Pagans S, Ortiz-Lombardia M, Espinas ML, Bernues J, Azorin F (2002) The Drosophila transcription factor tramtrack (TTK) interacts with Trithorax-like (GAGA) and represses GAGA-mediated activation. Nucleic Acids Res 30: 4406–4413. 12384587 10.1093/nar/gkf570 PMC137134

[32] Melnikova L, Juge F, Gruzdeva N, Mazur A, Cavalli G, Georgiev P. (2004) Interaction between the GAGA factor and Mod(mdg4) proteins promotes insulator bypass in Drosophila. Proc Natl Acad Sci U S A 101: 14806–14811. 10.1073/pnas.0403959101 15465920 PMC522021

[33] Schwendemann A, Lehmann M (2002) Pipsqueak and GAGA factor act in concert as partners at homeotic and many other loci. Proc Natl Acad Sci U S A 99: 12883–12888. 10.1073/pnas.202341499 12271134 PMC130554

[34] Faucheux M, Roignant JY, Netter S, Charollais J, Antoniewski C, Theodore L. (2003) batman Interacts with polycomb and trithorax group genes and encodes a BTB/POZ protein that is included in a complex containing GAGA factor. Mol Cell Biol 23: 1181–1195. 10.1128/MCB.23.4.1181-1195.2003 12556479 PMC141128

[35] Mishra K, Chopra VS, Srinivasan A, Mishra RK (2003) Trl-GAGA directly interacts with lola like and both are part of the repressive complex of Polycomb group of genes. Mech Dev 120: 681–689. 12834867 10.1016/s0925-4773(03)00046-7

[36] Espinas ML, Canudas S, Fanti L, Pimpinelli S, Casanova J, Azorin F. (2000) The GAGA factor of Drosophila interacts with SAP18, a Sin3-associated polypeptide. EMBO Rep 1: 253–259. 10.1093/embo-reports/kvd046 11256608 PMC1083720

[37] Erokhin M, Elizar'ev P, Parshikov A, Schedl P, Georgiev P, Chetverina D. (2015) Transcriptional read-through is not sufficient to induce an epigenetic switch in the silencing activity of Polycomb response elements. Proc Natl Acad Sci U S A 112: 14930–14935. 10.1073/pnas.1515276112 26504232 PMC4672805

[38] Kamakaka RT, Kadonaga JT (1994) The soluble nuclear fraction, a highly efficient transcription extract from Drosophila embryos. Methods Cell Biol 44: 225–235. 7707954 10.1016/s0091-679x(08)60916-4

[39] Shaposhnikov AV, Lebedeva LA, Chernioglo ES, Kachaev ZM, Abdrakhmanov A, Shidlovskii YV. (2016) Preparation of nuclear protein extract from Drosophila melanogaster embryos for studying transcription factors. Bioorg Khim 42: 712–721.

[40] Wright K (1989) Antibodies a laboratory manual: By E Harlow and D Lane. pp 726. Cold Spring Harbor Laboratory. 1988. $50 ISBN 0-87969-314-2. Biochemical Education 17: 220–220.

[41] Wolters DA, Washburn MP, Yates JR 3rd (2001) An automated multidimensional protein identification technology for shotgun proteomics. Anal Chem 73: 5683–5690. 11774908 10.1021/ac010617e

[42] McDonald WH, Tabb DL, Sadygov RG, MacCoss MJ, Venable J, Graumann J, et al (2004) MS1, MS2, and SQT-three unified, compact, and easily parsed file formats for the storage of shotgun proteomic spectra and identifications. Rapid Commun Mass Spectrom 18: 2162–2168. 10.1002/rcm.1603 15317041

[43] Peng J, Elias JE, Thoreen CC, Licklider LJ, Gygi SP (2003) Evaluation of multidimensional chromatography coupled with tandem mass spectrometry (LC/LC-MS/MS) for large-scale protein analysis: the yeast proteome. J Proteome Res 2: 43–50. 12643542 10.1021/pr025556v

[44] Xu T, Park SK, Venable JD, Wohlschlegel JA, Diedrich JK, Cociorva D, et al (2015) ProLuCID: An improved SEQUEST-like algorithm with enhanced sensitivity and specificity. J Proteomics 129: 16–24. 10.1016/j.jprot.2015.07.001 26171723 PMC4630125

[45] Aoki T, Schweinsberg S, Manasson J, Schedl P (2008) A stage-specific factor confers Fab-7 boundary activity during early embryogenesis in Drosophila. Mol Cell Biol 28: 1047–1060. 10.1128/MCB.01622-07 18039839 PMC2223392

[46] Wolle D, Cleard F, Aoki T, Deshpande G, Schedl P, Karch F. (2015) Functional Requirements for Fab-7 Boundary Activity in the Bithorax Complex. Mol Cell Biol 35: 3739–3752. 10.1128/MCB.00456-15 26303531 PMC4589599

[47] Mohrmann L, Langenberg K, Krijgsveld J, Kal AJ, Heck AJ, Verrijzer CP. (2004) Differential targeting of two distinct SWI/SNF-related Drosophila chromatin-remodeling complexes. Mol Cell Biol 24: 3077–3088. 10.1128/MCB.24.8.3077-3088.2004 15060132 PMC381637

[48] Chalkley GE, Moshkin YM, Langenberg K, Bezstarosti K, Blastyak A, Gyurkovics H, et al (2008) The transcriptional coactivator SAYP is a trithorax group signature subunit of the PBAP chromatin remodeling complex. Mol Cell Biol 28: 2920–2929. 10.1128/MCB.02217-07 18299390 PMC2293093

[49] Guruharsha KG, Rual JF, Zhai B, Mintseris J, Vaidya P, Beekman C, et al (2011) A protein complex network of Drosophila melanogaster. Cell 147: 690–703. 10.1016/j.cell.2011.08.047 22036573 PMC3319048

[50] Tsukiyama T, Daniel C, Tamkun J, Wu C (1995) ISWI, a member of the SWI2/SNF2 ATPase family, encodes the 140 kDa subunit of the nucleosome remodeling factor. Cell 83: 1021–1026. 8521502 10.1016/0092-8674(95)90217-1

[51] Kugler SJ, Nagel AC (2010) A novel Pzg-NURF complex regulates Notch target gene activity. Mol Biol Cell 21: 3443–3448. 10.1091/mbc.E10-03-0212 20685964 PMC2947479

[52] Ito T, Bulger M, Pazin MJ, Kobayashi R, Kadonaga JT (1997) ACF, an ISWI-containing and ATP-utilizing chromatin assembly and remodeling factor. Cell 90: 145–155. 9230310 10.1016/s0092-8674(00)80321-9

[53] Emelyanov AV, Vershilova E, Ignatyeva MA, Pokrovsky DK, Lu X, Konev AY, et al (2012) Identification and characterization of ToRC, a novel ISWI-containing ATP-dependent chromatin assembly complex. Genes Dev 26: 603–614. 10.1101/gad.180604.111 22426536 PMC3315121

[54] Reddy BA, Bajpe PK, Bassett A, Moshkin YM, Kozhevnikova E, Bezstarosti K, et al (2010) Drosophila transcription factor Tramtrack69 binds MEP1 to recruit the chromatin remodeler NuRD. Mol Cell Biol 30: 5234–5244. 10.1128/MCB.00266-10 20733004 PMC2953047

[55] Murawsky CM, Brehm A, Badenhorst P, Lowe N, Becker PB, Travers AA. (2001) Tramtrack69 interacts with the dMi-2 subunit of the Drosophila NuRD chromatin remodelling complex. EMBO Rep 2: 1089–1094. 10.1093/embo-reports/kve252 11743021 PMC1084170

[56] Chavez S, Beilharz T, Rondon AG, Erdjument-Bromage H, Tempst P, Svejstrup JQ, et al (2000) A protein complex containing Tho2, Hpr1, Mft1 and a novel protein, Thp2, connects transcription elongation with mitotic recombination in Saccharomyces cerevisiae. EMBO J 19: 5824–5834. 10.1093/emboj/19.21.5824 11060033 PMC305808

[57] Jimeno S, Rondon AG, Luna R, Aguilera A (2002) The yeast THO complex and mRNA export factors link RNA metabolism with transcription and genome instability. EMBO J 21: 3526–3535. 10.1093/emboj/cdf335 12093753 PMC126085

[58] Kopytova DV, Orlova AV, Krasnov AN, Gurskiy DY, Nikolenko JV, Nabirochkina EN, et al (2010) Multifunctional factor ENY2 is associated with the THO complex and promotes its recruitment onto nascent mRNA. Genes Dev 24: 86–96. 10.1101/gad.550010 20048002 PMC2802194

[59] Strasser K, Masuda S, Mason P, Pfannstiel J, Oppizzi M, Rodriguez-Navarro S, et al (2002) TREX is a conserved complex coupling transcription with messenger RNA export. Nature 417: 304–308. 10.1038/nature746 11979277

[60] Orphanides G, LeRoy G, Chang CH, Luse DS, Reinberg D (1998) FACT, a factor that facilitates transcript elongation through nucleosomes. Cell 92: 105–116. 9489704 10.1016/s0092-8674(00)80903-4

[61] Bauer M, Trupke J, Ringrose L (2016) The quest for mammalian Polycomb response elements: are we there yet? Chromosoma 125: 471–496. 10.1007/s00412-015-0539-4 26453572 PMC4901126

[62] Grossniklaus U, Paro R (2014) Transcriptional silencing by polycomb-group proteins. Cold Spring Harb Perspect Biol 6: a019331 10.1101/cshperspect.a019331 25367972 PMC4413232

[63] Kassis JA, Brown JL (2013) Polycomb group response elements in Drosophila and vertebrates. Adv Genet 81: 83–118. 10.1016/B978-0-12-407677-8.00003-8 23419717 PMC4157523

[64] McElroy KA, Kang H, Kuroda MI (2014) Are we there yet? Initial targeting of the Male-Specific Lethal and Polycomb group chromatin complexes in Drosophila. Open Biol 4: 140006 10.1098/rsob.140006 24671948 PMC3971409

[65] Schwartz YB, Pirrotta V (2013) A new world of Polycombs: unexpected partnerships and emerging functions. Nat Rev Genet 14: 853–864. 10.1038/nrg3603 24217316

[66] Orsi GA, Kasinathan S, Hughes KT, Saminadin-Peter S, Henikoff S, Ahmad K. (2014) High-resolution mapping defines the cooperative architecture of Polycomb response elements. Genome Res 24: 809–820. 10.1101/gr.163642.113 24668908 PMC4009610

[67] Ray P, De S, Mitra A, Bezstarosti K, Demmers JA, Pfeifer K, et al (2016) Combgap contributes to recruitment of Polycomb group proteins in Drosophila. Proc Natl Acad Sci U S A 113: 3826–3831. 10.1073/pnas.1520926113 27001825 PMC4833261

[68] Strutt H, Cavalli G, Paro R (1997) Co-localization of Polycomb protein and GAGA factor on regulatory elements responsible for the maintenance of homeotic gene expression. EMBO J 16: 3621–3632. 10.1093/emboj/16.12.3621 9218803 PMC1169986

[69] Gambetta MC, Oktaba K, Muller J (2009) Essential role of the glycosyltransferase sxc/Ogt in polycomb repression. Science 325: 93–96. 10.1126/science.1169727 19478141

[70] Ruhf ML, Braun A, Papoulas O, Tamkun JW, Randsholt N, Meister M. (2001) The domino gene of Drosophila encodes novel members of the SWI2/SNF2 family of DNA-dependent ATPases, which contribute to the silencing of homeotic genes. Development 128: 1429–1441. 11262242 10.1242/dev.128.8.1429

[71] Poux S, Melfi R, Pirrotta V (2001) Establishment of Polycomb silencing requires a transient interaction between PC and ESC. Genes Dev 15: 2509–2514. 10.1101/gad.208901 11581156 PMC312795

[72] Kellner WA, Van Bortle K, Li L, Ramos E, Takenaka N, Corces VG. (2013) Distinct isoforms of the Drosophila Brd4 homologue are present at enhancers, promoters and insulator sites. Nucleic Acids Res 41: 9274–9283. 10.1093/nar/gkt722 23945939 PMC3814382

[73] Crosby MA, Miller C, Alon T, Watson KL, Verrijzer CP, Goldman-Levi R, et al (1999) The trithorax group gene moira encodes a brahma-associated putative chromatin-remodeling factor in Drosophila melanogaster. Mol Cell Biol 19: 1159–1170. 9891050 10.1128/mcb.19.2.1159 PMC116045

[74] Dingwall AK, Beek SJ, McCallum CM, Tamkun JW, Kalpana GV, Goff SP, et al (1995) The Drosophila snr1 and brm proteins are related to yeast SWI/SNF proteins and are components of a large protein complex. Mol Biol Cell 6: 777–791. 7579694 10.1091/mbc.6.7.777 PMC301240

[75] Kennison JA, Tamkun JW (1988) Dosage-dependent modifiers of polycomb and antennapedia mutations in Drosophila. Proc Natl Acad Sci U S A 85: 8136–8140. 3141923 10.1073/pnas.85.21.8136 PMC282375

[76] Chang YL, King B, Lin SC, Kennison JA, Huang DH (2007) A double-bromodomain protein, FSH-S, activates the homeotic gene ultrabithorax through a critical promoter-proximal region. Mol Cell Biol 27: 5486–5498. 10.1128/MCB.00692-07 17526731 PMC1952094

[77] Shearn A (1989) The ash-1, ash-2 and trithorax genes of Drosophila melanogaster are functionally related. Genetics 121: 517–525. 2497049 10.1093/genetics/121.3.517 PMC1203637

[78] Gildea JJ, Lopez R, Shearn A (2000) A screen for new trithorax group genes identified little imaginal discs, the Drosophila melanogaster homologue of human retinoblastoma binding protein 2. Genetics 156: 645–663. 11014813 10.1093/genetics/156.2.645 PMC1461290

[79] Fereres S, Simon R, Mohd-Sarip A, Verrijzer CP, Busturia A (2014) dRYBP counteracts chromatin-dependent activation and repression of transcription. PLoS One 9: e113255 10.1371/journal.pone.0113255 25415640 PMC4240632

[80] Belozerov VE, Majumder P, Shen P, Cai HN (2003) A novel boundary element may facilitate independent gene regulation in the Antennapedia complex of Drosophila. EMBO J 22: 3113–3121. 10.1093/emboj/cdg297 12805225 PMC162149

[81] Schweinsberg S, Hagstrom K, Gohl D, Schedl P, Kumar RP, Mishra R, et al (2004) The enhancer-blocking activity of the Fab-7 boundary from the Drosophila bithorax complex requires GAGA-factor-binding sites. Genetics 168: 1371–1384. 10.1534/genetics.104.029561 15579691 PMC1448804

[82] Schweinsberg SE, Schedl P (2004) Developmental modulation of Fab-7 boundary function. Development 131: 4743–4749. 10.1242/dev.01343 15329342

[83] Pai CY, Lei EP, Ghosh D, Corces VG (2004) The centrosomal protein CP190 is a component of the gypsy chromatin insulator. Mol Cell 16: 737–748. 10.1016/j.molcel.2004.11.004 15574329

[84] Gause M, Morcillo P, Dorsett D (2001) Insulation of enhancer-promoter communication by a gypsy transposon insert in the Drosophila cut gene: cooperation between suppressor of hairy-wing and modifier of mdg4 proteins. Mol Cell Biol 21: 4807–4817. 10.1128/MCB.21.14.4807-4817.2001 11416154 PMC87172

[85] Ghosh D, Gerasimova TI, Corces VG (2001) Interactions between the Su(Hw) and Mod(mdg4) proteins required for gypsy insulator function. EMBO J 20: 2518–2527. 10.1093/emboj/20.10.2518 11350941 PMC125459

[86] Zollman S, Godt D, Prive GG, Couderc JL, Laski FA (1994) The BTB domain, found primarily in zinc finger proteins, defines an evolutionarily conserved family that includes several developmentally regulated genes in Drosophila. Proc Natl Acad Sci U S A 91: 10717–10721. 7938017 10.1073/pnas.91.22.10717 PMC45093

[87] Cuartero S, Fresan U, Reina O, Planet E, Espinas ML (2014) Ibf1 and Ibf2 are novel CP190-interacting proteins required for insulator function. EMBO J 33: 637–647. 10.1002/embj.201386001 24502977 PMC3989656

[88] Maksimenko O, Bartkuhn M, Stakhov V, Herold M, Zolotarev N, Jox T, et al (2015) Two new insulator proteins, Pita and ZIPIC, target CP190 to chromatin. Genome Res 25: 89–99. 10.1101/gr.174169.114 25342723 PMC4317163

