# Supplementary material for: The GAGA factor regulatory network: Identification of GAGA factor associated proteins
Source: PLoS One. 2017 Mar 15;12(3):e0173602. doi: 10.1371/journal.pone.0173602 (PMC5351981; doi:10.1371/journal.pone.0173602)
Supplement: S1 File — Supplementary figures A-E. (PDF) [file pone.0173602.s001.pdf]

Additional file S1 Supplementary Figures A-E

The GAGA factor regulatory network: Identification of GAGA factor associated proteins

Lomaev et al.

This file includes

Supplementary Figures A-E:

Figure A. Nuclear extracts prepared from 0-12 hr wild type embryos.
Figure B. Purification of GAF associated proteins: GAF IP 1.
Figure C. Purification of GAF associated proteins: GAF IP 2 and 3.
Figure D. GO analysis of GAF associated gene products with $p<0.05$.
Figure E. Co-IP of GAF and selected proteins.

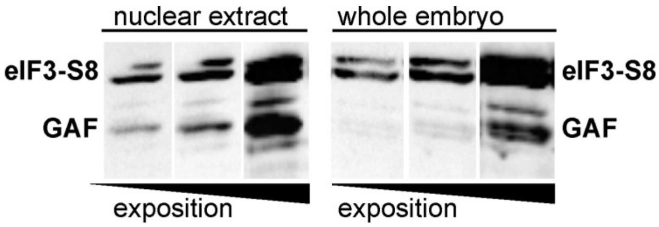

Figure A. Nuclear extracts prepared from 0-12 hr wild type embryos. The enrichment of GAF protein in nuclear extracts is shown. Western blot analysis of protein extracts isolated from nucleus (Left) or whole embryo (Right) was performed. Detection was performed simultaneously against GAF and eIF3-S8 cytoplasmic protein. Different exposition times are shown.

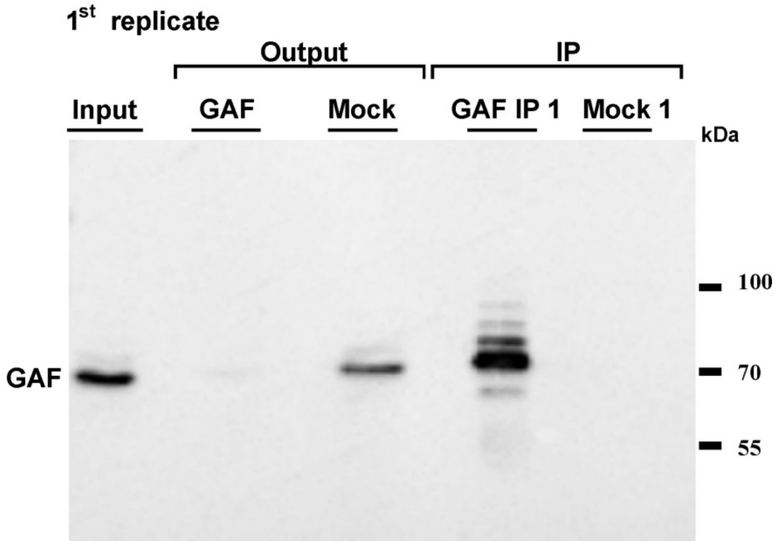

Figure B. Purification of GAF associated proteins: GAF IP 1. Western blot analysis of nuclear extracts (Input), unbound fractions (Output) or immunoprecipitates (IP) obtained with GAF coupled Protein A (GAF) or uncoupled Protein A sepharose (Mock) detected with antibodies against GAF. GAF IP 1 and Mock 1 probes are shown. 0.2% of Input and Output; 0.8% of IP were loaded onto the gel.

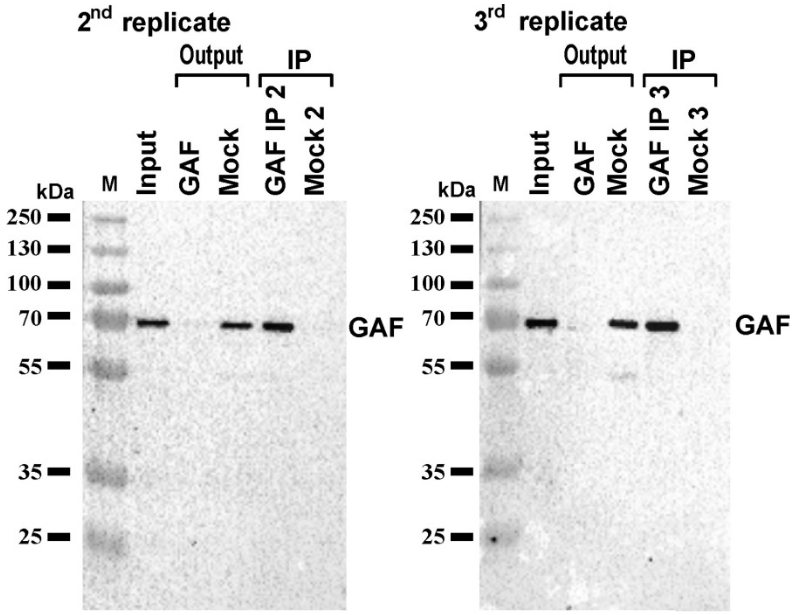

Figure C. Purification of GAF associated proteins: GAF IP 2 and 3. GAF IP 2,3 and Mock 2,3 probes are shown. Designations as in Suppl Figure 1. 0.2% of Input and Output; 0.375% of IP were loaded onto the gel.

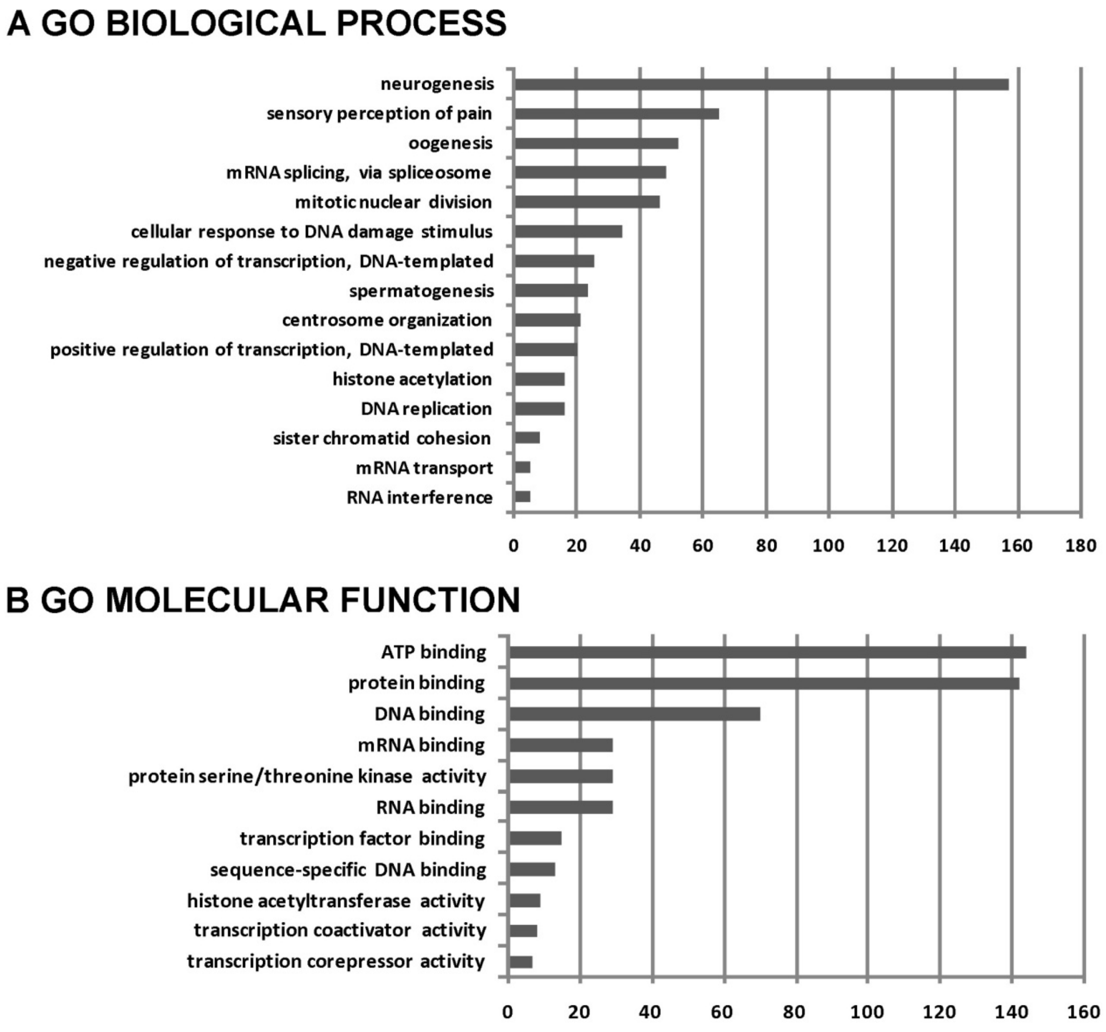

Figure D. GO analysis of GAF associated gene products with p<0.05. GO analysis was performed using Flybase. A. Biological process. B. Molecular function.

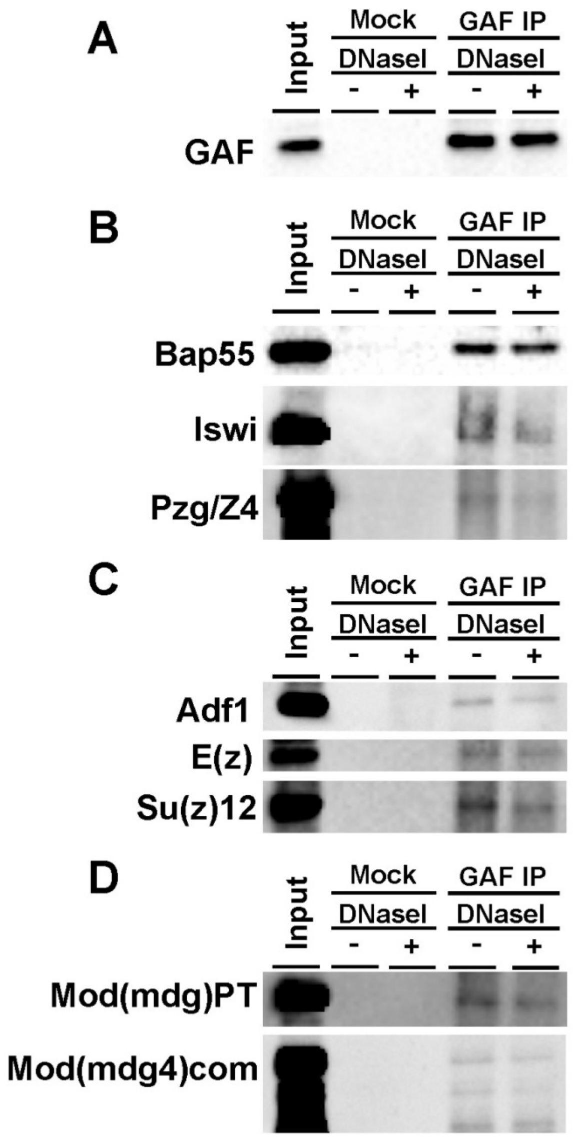

Figure E. Co-IP of GAF and selected proteins. Western blot analysis of embryo nuclear extracts (Input), control immunoprecipitates (Mock), and immunoprecipitates (GAF IP) are shown. Immunopreciitation was performed in the presence (+) or absence (-) of DNaseI. Antibodies used to detect GAF associated partners were as follows: (A) anti-GAF; (B) to chromatin remodelers, anti-Bap55, anti-Iswi, and NURF complex associated protein Pzg/Z4; (C) to Polycomb factors, anti-Adf1, anti-E(z) and anti-Su(z)12; (D) to chromosome architecture factors, anti-Mod(mdg4) specific to PT(67.2) isoform, anti-Mod(mdg4) common to different isoforms. 11% of Input and Output; 20% of IP were loaded onto the gel.
