# Supplementary material for: The GAGA factor regulatory network: Identification of GAGA factor associated proteins
Source: PLoS One. 2017 Mar 15;12(3):e0173602. doi: 10.1371/journal.pone.0173602 (PMC5351981; doi:10.1371/journal.pone.0173602)
Supplement: S9 File — (PDF) [file pone.0173602.s009.pdf]

```
                                                                                              Section 1
                        (1) 1        10        20        30        40        50        60        70          83
     Ab PB 53-197 aa    (1) GSALSPATPPPSLNLSHQQQQHQQHYALKWNDFQSSILSSFRHLRDEEDFVDVTLACDERSFTAHKVVLSACSPYFRRLLKAN
      GAF PC 1-131 aa  (1) -----------------MSLPMNSLYSLTWGDYGTSLVSAIQLLRCHGDLVDCTLAAGGRSFPAHKIVLCAASPFLLDLLKNT
        Lolal 1-127 aa (1) ------------------MMSSDQQFFLKWNDFQTNMVTSFRHLRDEKSFTDVTLACEGQTCKAHKMVLSACSPYFKALLEEN
  Bab2 PA 194-313 aa   (1) ---------------------EGQQFCLRWNNYQSNLTNVFDELLQSESFVDVTLSCEGHSIKAHKMVLSACSPYFQALFYDN
   Psq/BTB V 1-141 aa  (1) ----------------MAAVRGHQYFSLRWNNYQNTMTSVFQQLREDLSFVDVTLSCEHGSLKAHKVVLSACSTYFQKLLLEN
     CG8924 PB 1-146 aa (1) ------------------MSGATQEFCVRWNSHLGSIGAAFPQLLAGQRFVDVTLACEGQQVHCHRLVLAACSTYFEAILAEH
    Mod(mdg4) 1-131 aa  (1) -------------------MADDEQFSLCWNNFNTNLSAGFHESLCRGDLVDVSLAAEGQIVKAHRLVLSVCSPFFRKMFTQM
      Lola PA 1-120 aa  (1) -------------------MDDDQQFCLRWNNHQSTLISVFDTLLENETLVDCTLAAEGKFLKAHKVVLSACSPYFATLLQEQ
    BtbVII PA 1-119 aa  (1) --------------------MSVQQFCLRWNNHQPNFISVCSSLLHNGTLVDVTLAAEGRQLQAHKIVLSACSSYFQALFTTN
           Ttk 1-131 aa  (1) ------------------MKMASQRFCLRWNNHQSNLLSVFDQLLHAETFTDVTLAVEGQHLKAHKMVLSACSPYFNTLFVSH
                                                                                              Section 2
                       (84) 84   90        100       110       120       130       140       150          164
     Ab PB 53-197 aa   (84) P-CEHPIVILRDVRCDDVENLLSFMYNGEVNVSHEQLPDFLKTAHLLQIRGLADVNGGYPYSK------------------
      GAF PC 1-131 aa  (67) P-CKHPVVMLAGVNANDLEALLEFVYRGEVSVDHAQLPSLLQAAQCLNIQGLAPQTVTKDDYTTHS---------------
        Lolal 1-127 aa (66) P-SKHPIIILKDVSYIHLQAILEFMYAGEVNVSQEQLPAFLKTADRLKVKGLAETPSSIKREG------------------
  Bab2 PA 194-313 aa  (63) PCQHP-IIIMRDVSWSDLKALVEFMYKGEINVCQDQINPLLKVAETLKIRGLAEVSAGR----------------------
   Psq/BTB V 1-141 aa (68) PCKHPTIILPADIIFTDLKTIIDFVYRGEIDVTESELQGLLRTAEQLKIKGLCETAENADDLNDAATATITVSE-------
     CG8924 PB 1-146 aa (66) PCKHPVIILPREIKLWEIQALVDFMYKGEVNVTQAGLGQLLRCAEQLQIRGLYGSEAPINYKKLQQASLEAAKDPAATTAR
    Mod(mdg4) 1-131 aa (65) PSNTHAIVFLNNVSHSALKDLIQFMYCGEVNVKQDALPAFISTAESLQIKGLTDNDPAPQPPQESSP--------------
      Lola PA 1-120 aa  (65) Y-DKHPIFILKDVKYQELRAMMDYMYRGEVNISQDQLAALLKAAESLQIKGLSDNRT------------------------
    BtbVII PA 1-119 aa (64) P-CQHPIVILKDVQYDDLKTMVDFMYYGEVNVSQEQLPHILKTAEMLKIKGLAEMPT------------------------
           Ttk 1-131 aa (66) P-EKHPIVILKDVPYSDMKSLLDFMYRGEVSVDQERLTAFLRVAESLRIKGLTEVNDDKPSPAAAAA--------------
```

CG8924 BTB alignment with BTB domains of known Ttk-group members (Ab, GAF, Batman (Lolal), Bab2, Psq (BTBV), Mod(mdg4), Lola, BtbVII, Ttk).

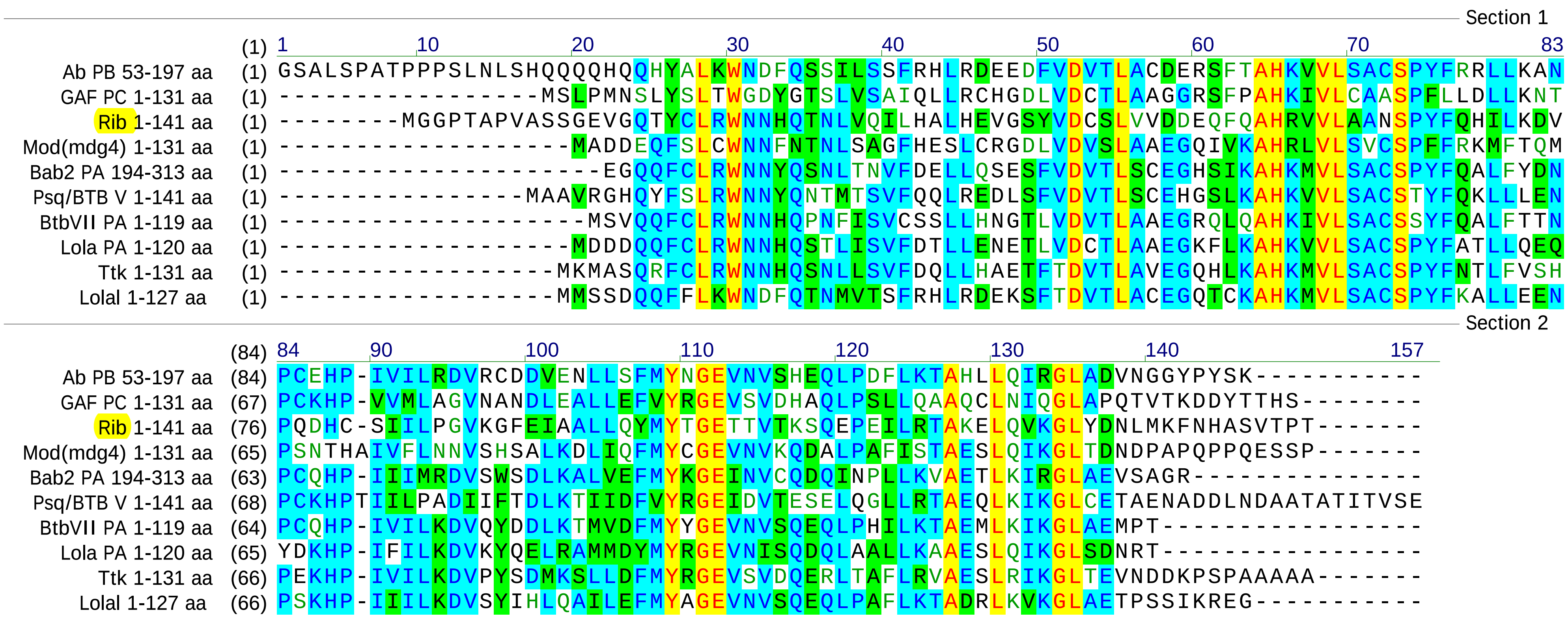

Rib BTB alignment with BTB domains of Ttk-group members

```
                                                                                                   Section 1
                     (1) 1        10        20        30        40        50        60        70          83
      Ab PB 53-197 aa (1) GSALSPATPPPSLNLSHQQQQHQQHYALKWNDFQSSILSSFRHLRDEEDFVDVTLACDE-RSFTAHKVVLSACSPYFRRLLKA
      GAF PC 1-131 aa (1) -----------------MSLPMNSLYSLTWGDYGTSLVSAIQLLRCHGDLVDCTLAAGG-RSFPAHKIVLCAASPFLLDLLKN
     CP190 PA 1-131 aa (1) ---------------------MGEVKSVKVDNWGVFFLQKLQNFFNKTDYCDLTLQFRDNSQLKVHRLVLSACTDYFNVLEQT
    Psq/BTB V 1-141 aa (1) ----------------MAAVRGHQYFSLRWNNYQNTMTSVFQQLREDLSFVDVTLSCEH-GSLKAHKVVLSACSTYFQKLLE
       Mod(mdg4) 1-131 aa (1) -------------------MADDEQFSLCWNNFNTNLSAGFHESLCRGDLVDVSLAAEG-QIVKAHRLVLSVCSPFFRKMFTQ
   Bab2 PA 194-313 aa (1) ---------------------EGQQFCLRWNNYQSNLTNVFDELLQSESFVDVTLSCEG-HSIKAHKMVLSACSPYFQALFYD
     BtbVII PA 1-119 aa (1) --------------------MSVQQFCLRWNNHQPNFISVCSSLLHNGTLVDVTLAAEG-RQLQAHKIVLSACSSYFQALFTT
        Lola PA 1-120 aa (1) -------------------MDDDQQFCLRWNNHQSTLISVFDTLLENETLVDCTLAAEG-KFLKAHKVVLSACSPYFATLLQE
            Ttk 1-131 aa (1) ------------------MKMASQRFCLRWNNHQSNLLSVFDQLLHAETFTDVTLAVEG-QHLKAHKMVLSACSPYFNTLFVS
          Lolal 1-127 aa (1) ------------------MMSSDQQFFLKWNDFQTNMVTSFRHLRDEKSFTDVTLACEG-QTCKAHKMVLSACSPYFKALLEE
                                                                                                   Section 2
                    (84) 84   90        100       110       120       130       140               158
      Ab PB 53-197 aa (83) NPC-EHPIVILRDVRCDDVENLLSFMYNGEVNVSHEQLPDFLKTAHLLQIRGLADVNGGYPYSK-----------
      GAF PC 1-131 aa (66) TPC-KHPVVMLAGVNANDLEALLEFVYRGEVSVDHAQLPSLLQAAQCLNIQGLAPQTVTKDDYTTHS--------
     CP190 PA 1-131 aa (63) CEIVDDALIMPNEFQADVVVPIVNFMYTGTLEFELKMYGKLLRTAKEMNMTVLLKLLEAHRRTMENVNR------
    Psq/BTB V 1-141 aa (67) NPCKHPTIILPADIIFTDLKTIIDFVYRGEIDVTESELQGLLRTAEQLKIKGLCETAENADDLNDAATATITVSE
       Mod(mdg4) 1-131 aa (64) MPSNTHAIVFLNNVSHSALKDLIQFMYCGEVNVKQDALPAFISTAESLQIKGLTDNDPAPQPPQESSP-------
   Bab2 PA 194-313 aa (62) NPC-QHPIIIMRDVSWSDLKALVEFMYKGEINVCQDQINPLLKVAETLKIRGLAEVSAGR---------------
     BtbVII PA 1-119 aa (63) NPC-QHPIVILKDVQYDDLKTMVDFMYYGEVNVSQEQLPHILKTAEMLKIKGLAEMPT-----------------
        Lola PA 1-120 aa (64) QYD-KHPIFILKDVKYQELRAMMDYMYRGEVNISQDQLAALLKAAESLQIKGLSDNRT-----------------
            Ttk 1-131 aa (65) HPE-KHPIVILKDVPYSDMKSLLDFMYRGEVSVDQERLTAFLRVAESLRIKGLTEVNDDKPSPAAAAA-------
          Lolal 1-127 aa (65) NPS-KHPIIILKDVSYIHLQAILEFMYAGEVNVSQEQLPAFLKTADRLKVKGLAETPSSIKREG-----------
```

CP190 BTB alignment with BTB domains of Ttk-group members

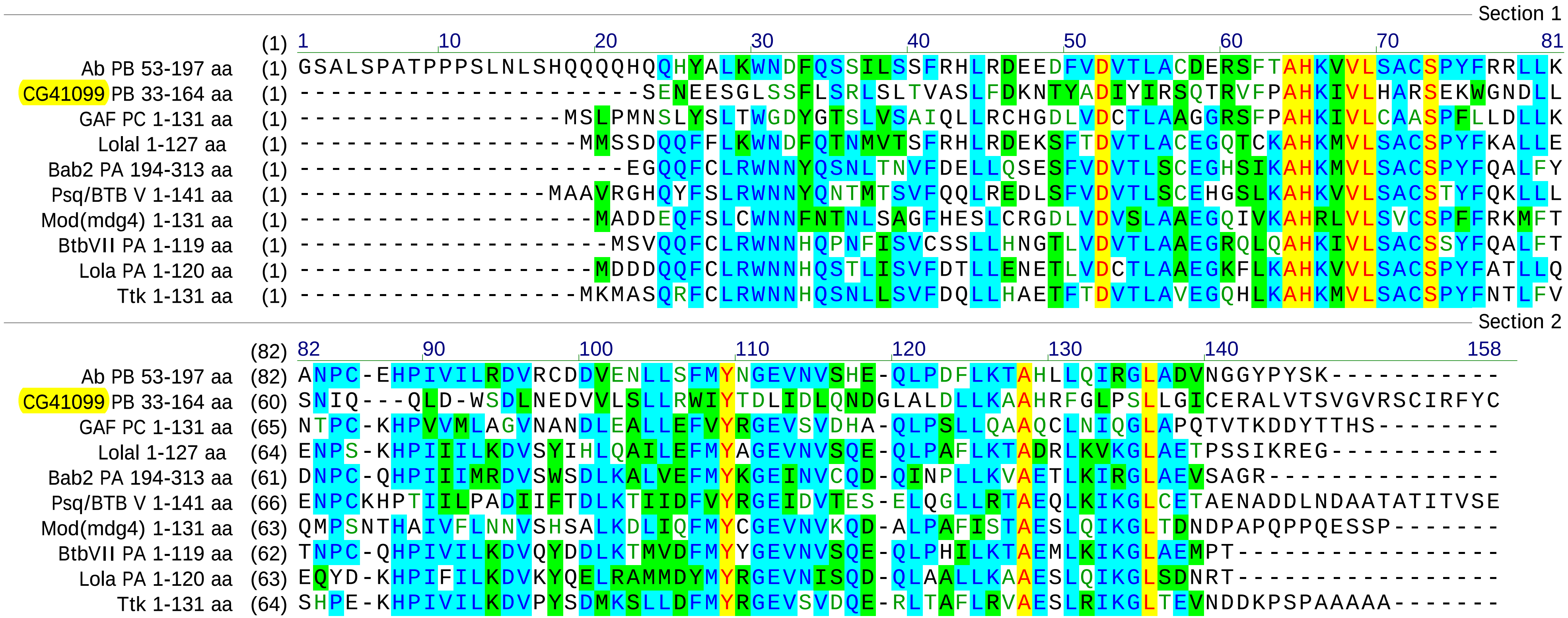

CG41099 BTB alignment with BTB domains of Ttk-group members

Section 1

```
                          (1)  1        10        20        30        40        50        60        70          83
Ab PB 53-197 aa           (1)  GSALSPATPPPSLNLSHQQQQHQQHYALKWNDFQSSILSSFRHLRDEEDFVDVTLACDERSFTAHKVVLSACSPYFRRLLKAN
Lolal 1-127 aa            (1)  ------------------MMSSDQQFFLKWNDFQTNMVTSFRHLRDEKSFTDVTLACEGQTCKAHKMVLSACSPYFKALLEEN
GAF PC 1-131 aa           (1)  -----------------MSLPMNSLYSLTWGDYGTSLVSAIQLLRCHGDLVDCTLAAGGRSFPAHKIVLCAASPFLLDLLKNT
Mri PA 86-210 aa          (1)  ----------------------------------SGMGRLPLPPERITMLVDGVRFTVEQSLLTAHPTTMLGTMFGSGFQFAH
Mod(mdg4) 1-131 aa        (1)  -------------------MADDEQFSLCWNNFNTNLSAGFHESLCRGDLVDVSLAAEGQIVKAHRLVLSVCSPFFRKMFTQM
Bab2 PA 194-313 aa        (1)  ---------------------EGQQFCLRWNNYQSNLTNVFDELLQSESFVDVTLSCEGHSIKAHKMVLSACSPYFQALFYDN
Psq/BTB V 1-141 aa        (1)  ----------------MAAVRGHQYFSLRWNNYQNTMTSVFQQLREDLSFVDVTLSCEHGSLKAHKVVLSACSTYFQKLLLEN
BtbVII PA 1-119 aa        (1)  --------------------MSVQQFCLRWNNHQPNFISVCSSLLHNGTLVDVTLAAEGRQLQAHKIVLSACSSYFQALFTTN
Lola PA 1-120 aa          (1)  -------------------MDDDQQFCLRWNNHQSTLISVFDTLLENETLVDCTLAAEGKFLKAHKVVLSACSPYFATLLQEQ
Ttk 1-131 aa              (1)  ------------------MKMASQRFCLRWNNHQSNLLSVFDQLLHAETFTDVTLAVEGQHLKAHKMVLSACSPYFNTLFVSH
```

Section 2

```
                         (84)  84    90        100       110       120       130       140       150            166
Ab PB 53-197 aa          (84)  PCEHP-IVILRDVRCDDVENLLSFMYNGEVNVSHEQ-------------LPDFLKTAHLLQIRGLADVNGGYPYSK-------
Lolal 1-127 aa           (66)  PSKHP-IIILKDVSYIHLQAILEFMYAGEVNVSQEQ-------------LPAFLKTADRLKVKGLAETPSSIKREG-------
GAF PC 1-131 aa          (67)  PCKHP-VVMLAGVNANDLEALLEFVYRGEVSVDHAQ-------------LPSLLQAAQCLNIQGLAPQTVTKDDYTTHS----
Mri PA 86-210 aa         (50)  TNERGEYDVADGISHLVFRAILEYYKSGVIRCPPTVSVPELKEACDYLLIPFDATTVRCQNLRGLLHELSNEGARQ-------
Mod(mdg4) 1-131 aa       (65)  PSNTHAIVFLNNVSHSALKDLIQFMYCGEVNVKQDA-------------LPAFISTAESLQIKGLTDNDPAPQPPQESSP---
Bab2 PA 194-313 aa       (63)  PCQHP-IIIMRDVSWSDLKALVEFMYKGEINVCQDQ-------------INPLLKVAETLKIRGLAEVSAGR-----------
Psq/BTB V 1-141 aa       (68)  PCKHPTIILPADIIFTDLKTIIDFVYRGEIDVTESE-------------LQGLLRTAEQLKIKGLCETAENADDLNDAATATI
BtbVII PA 1-119 aa       (64)  PCQHP-IVILKDVQYDDLKTMVDFMYYGEVNVSQEQ-------------LPHILKTAEMLKIKGLAEMPT-------------
Lola PA 1-120 aa         (65)  YDKHP-IFILKDVKYQELRAMMDYMYRGEVNISQDQ-------------LAALLKAAESLQIKGLSDNRT-------------
Ttk 1-131 aa             (66)  PEKHP-IVILKDVPYSDMKSLLDFMYRGEVSVDQER-------------LTAFLRVAESLRIKGLTEVNDDKPSPAAAAA---
```

Mri BTB alignment with BTB domains of Ttk-group members
